# Mechanism of RNA polymerase I selection by transcription factor UAF

**DOI:** 10.1126/sciadv.abn5725

**Published:** 2022-04-20

**Authors:** Florence Baudin, Brice Murciano, Herman K. H. Fung, Simon A. Fromm, Simone Mattei, Julia Mahamid, Christoph W. Müller

**Affiliations:** 1 Structural and Computational Biology Unit, European Molecular Biology Laboratory, Heidelberg, Germany.; 2 EMBL Imaging Centre, European Molecular Biology Laboratory, Heidelberg, Germany.

## Abstract

Preribosomal RNA is selectively transcribed by RNA polymerase (Pol) I in eukaryotes. The yeast transcription factor upstream activating factor (UAF) represses Pol II transcription and mediates Pol I preinitiation complex (PIC) formation at the 35*S* ribosomal RNA gene. To visualize the molecular intermediates toward PIC formation, we determined the structure of UAF in complex with native promoter DNA and transcription factor TATA-box-binding protein (TBP). We found that UAF recognizes DNA using a hexameric histone-like scaffold with markedly different interactions compared with the nucleosome and the histone-fold-rich transcription factor IID (TFIID). In parallel, UAF positions TBP for Core Factor binding, which leads to Pol I recruitment, while sequestering it from DNA and Pol II/III–specific transcription factors. Our work thus reveals the structural basis of RNA Pol selection by a transcription factor.

## Teaser

UAF uses a histone-like hexamer for DNA recognition and Pol I–selective positioning of TBP at the 35*S* rRNA gene promoter.

## INTRODUCTION

A unique set of transcription factors act to selectively recruit RNA polymerase (Pol) I for the transcription of preribosomal RNA in eukaryotes (*1*). These factors work in tandem to establish a Pol I preinitiation complex (PIC) at the target gene promoter. The budding yeast transcription factor upstream activating factor (UAF) is essential for repressing Pol II transcription at the 35*S* ribosomal RNA (rRNA) gene promoter (*2*). Disruption of UAF leads to Pol II recruitment and transcription of 35*S* rRNA by Pol II instead of I (*2*–*5*). Binding 41 to 155 base pairs (bp) upstream of the transcription start site (*6*–*9*), UAF forms a complex with transcription factors TATA-box-binding protein (TBP) and Core Factor and initiation factor Rrn3 to establish the Pol I PIC (*10*). Hence, UAF serves a parallel but apparently mutually exclusive role to transcriptional regulators TFIID and SAGA, which catalyze Pol II PIC formation, and TFIIIB, which catalyzes Pol III PIC formation (*11*–*13*). TFIID, Spt-Ada-Gcn5 acetyltransferase (SAGA), and TFIIIB all stabilize TBP at their target genes. During Pol II PIC assembly, TBP is subsequently handed over to TFIIA and TFIIB for transcription initiation. It is yet unclear why Pol I is selectively recruited to the 35*S* rRNA gene promoter despite the presence of TBP. The organization of TBP with respect to DNA, UAF, and Core Factor has remained elusive. It has been speculated that TBP does not contact DNA directly but rather bridges between UAF and Core Factor to support specific Rrn3 and Pol I recruitment (*10*). While the structure of a minimal transcriptionally active complex comprising Core Factor, Rrn3, and Pol I has been determined (*14*–*16*), the structure of UAF, its promoter DNA recognition mechanisms, and interaction surfaces with TBP have remained unelucidated.

A six-component protein complex, UAF comprises subunits Rrn5, Rrn9, Rrn10, Uaf30, and histones H3 and H4 (*17*, *18*). By native mass spectrometry, it has been found that UAF contains two H3 proteins and one H4 protein (*19*). Further modeling based on cross-linking mass spectrometry and domain predictions suggests that subunit Rrn5 interacts with H3 and H4 to form a H3-H4 tetramer–like core that is surrounded by subunits Rrn9, Rrn10, and Uaf30 (*20*). Also found in TFIID, SAGA, and negative cofactor 2 (NC2), the histone fold appears to be a recurring motif in transcriptional regulators (*21*–*23*), although whether and how it contributes to DNA binding in UAF are to date unknown. While homologs of UAF have been identified in several fungal species (*24*), the upstream rRNA promoter region in metazoans is occupied by the protein upstream binding factor (UBF), which has a different domain architecture, whereas TBP is recruited as part of a larger Core Factor–like complex, selectivity factor 1 (SL1), during Pol I PIC assembly (*1*). Comprising multiple high-mobility group box domains, UBF has a much larger genomic footprint than UAF that is on the kilobase scale (*9*, *25*). Thus, to elucidate the structure of yeast UAF, and to understand how it binds DNA at a defined site and interacts with TBP, we reconstituted a complex between UAF, native promoter DNA, and TBP for analysis. Using cryo–electron microscopy (cryo-EM), we determined the structure of the complex and found that UAF uses its histone fold–based structure to recognize DNA and that it interacts with a conserved regulatory surface of TBP while sequestering it from DNA to enable specific Pol I recruitment. Thus, here, we reveal the molecular principles underlying UAF function.

## RESULTS

### Structure of the UAF-TBP-DNA complex reveals a histone-like core in UAF

**Fig. 1.**
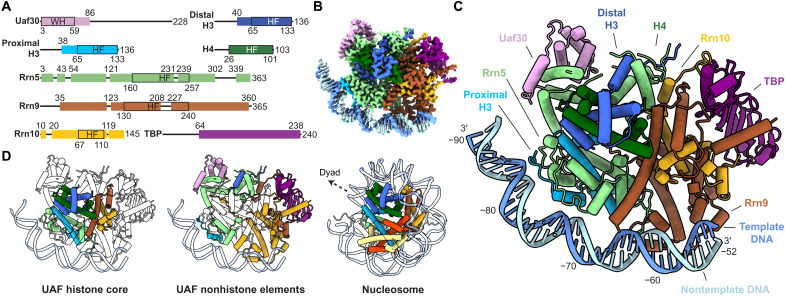
Structure of UAF with TBP and promoter DNA. (**A**) Domain organization of UAF subunits. Residues built are represented as colored boxes with ranges indicated on top and domain boundaries (black outlines) indicated below. WH, winged helix; HF, histone fold. Loops L1 and L2 of the Rrn5 HF, helices ɑ1 to ɑ2 of the Rrn9 HF are extended relative to histone proteins H4 and H2B, respectively. The Rrn10 HF is missing the corresponding helix ɑ3 of H2A. (**B**) Cryo-EM density map of UAF, postprocessed with DeepEMhancer (*45*). (**C**) Atomic model represented as pipes (ɑ helices), planks (β strands), ribbons (DNA backbone), and sticks (DNA bases). The respective subunits are labeled and colored as in (A). (**D**) Histone-like hexameric core [left, HF domains outlined in (A)] and non–histone-like periphery (middle) of UAF, colored by subunit. The budding yeast nucleosome [Protein Data Bank (PDB), 1ID3] (*26*) is shown in the same orientation on the right, with the dyad axis indicated. Corresponding histones are colored as in UAF.

To reconstitute the ternary complex, we produced UAF recombinantly by coexpression of all subunits in *Escherichia coli* (Fig. 1A and fig. S1A) and incubated the complex with polymerase chain reaction (PCR)–amplified DNA spanning positions −190 to −40 with respect to the transcription start site and recombinant TBP. We observed that the addition of TBP and DNA helps to stabilize the structure of UAF, allowing it to be resolved more clearly by cryo-EM (fig. S1B). Classification and refinement of particles yielded a reconstruction at 2.8-Å resolution, where most of the ternary complex was resolved except for parts of Uaf30 and upstream DNA from positions −190 to −91 (Fig. 1, A to C; figs. S1, C and D, and S2; and table S1). Given the pattern of purines and pyrimidines in the DNA sequence and the quality of the density observed, we were able to determine the DNA register unambiguously (fig. S2C).

In the refined structure, UAF occupies positions −85 to −54 of the DNA, whereas TBP is held away from DNA by UAF. Notably, Rrn9, Rrn10, and Rrn5 all contain a histone fold, sharing homology with histones H2B, H2A, and H4, respectively (fig. S3, A and B). These folds combine with two H3 and one H4 to form a hexameric histone-like core within UAF (Fig. 1D). This architecture is consistent with previous predictions (*19*, *20*) and is reminiscent of transcription factor complex TFIID and transcriptional coactivator SAGA, which feature histone-like octamers and hexamers and perform a parallel function in support of Pol II PIC assembly (fig. S3C). Similar to TFIID and SAGA, non–histone-like elements decorate the UAF histone–like core to enable specific protein-protein interactions. In contrast to TFIID and the nucleosome, and as yet elusive for SAGA, the decorated UAF core also enables long-range specific protein-DNA interactions. Like so, Rrn9 and Rrn10 bind TBP and DNA downstream, and Rrn5 with a partner H3 binds DNA upstream. We hereafter refer to the DNA binding H3 as the proximal H3. Rrn5 additionally contains a SANT domain, which contacts Rrn10 and the distal H3, further stabilizing the UAF complex. Completing the assembly, Uaf30 joins Rrn5 at the upstream end of the Rrn5-H3-H4 tetramer. Three helices of the predicted N-terminal winged helix domain contact Rrn5, consistent with previous observations that the domain interfaces with other UAF subunits (*20*). The remainder of Uaf30 is unresolved, pointing to an inherent flexibility of the protein. However, upon further classification, a subpopulation of particles show density emanating from the Uaf30 region toward DNA at approximately position −96, suggesting that Uaf30 also has a role in contacting DNA (fig. S1C). Beyond position −100, there is little sign of DNA contacting UAF again either in the final reconstruction or in class averages of the full dataset (fig. S1C).

### The histone-like core drives promoter DNA recognition

**Fig. 2.**
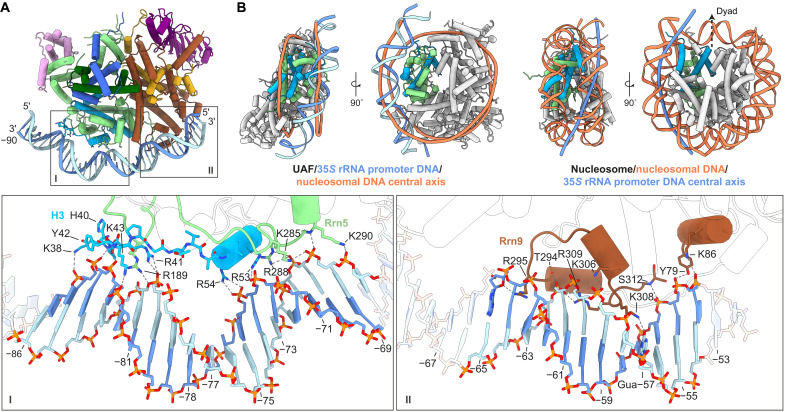
Interactions of UAF with promoter DNA. (**A**) Overview of the UAF-DNA interface. Insets show the chemical environments of the Rrn5-H3-contact site (I) and Rrn9 contact site (II), respectively. Participating secondary structure elements are colored. Bases in the promoter sequence relative to the 35*S* rRNA transcription start site are indicated. (**B**) 35*S* rRNA promoter DNA position relative to the nucleosome. UAF is aligned to the yeast nucleosome (PDB, 1ID3) (*26*) based on Rrn5 (green) and the proximal H3 (blue). Left: UAF-TBP-DNA superposed with the central axis of nucleosomal DNA (orange), calculated with Curves+ (*51*). Right: Corresponding views of the nucleosome. Superposed is the central axis of UAF-bound DNA (dark blue). The H3 (cyan) and H4 (green) subunits used for alignment with UAF are colored, and the dyad axis is indicated.

Despite the presence of histone folds, UAF does not contact promoter DNA using canonical nucleosome interactions. Rather, a distinctly curved, positively charged surface, formed by a combination of histone folds and nonhistone elements, serves as a DNA binding surface (Fig. 2A and fig. S4A). Correspondingly, bound DNA is distinctly bent.

At the Rrn5-H3 contact site, spanning positions −86 to −68, only the N-terminal loop and N-terminal helix of H3 contact DNA, unlike in the nucleosome, where the rest of the H3 histone fold also engages with DNA. A conserved arginine (*26*) Arg^189^ of loop L1 of the Rrn5 histone fold contacts DNA. However, all other contacts by Rrn5 occur C terminal to the histone fold. Consequently, bound DNA is translated ~10 Å away from its canonical position compared with the nucleosome (Fig. 2B). The bound DNA is bent strongly around position −78/−77, where a thymine-adenine (TA) dinucleotide occurs, with compression of the major groove on the protein-facing side and widening of the minor groove opposite (fig. S5A). Like in the nucleosome, this bend is stabilized by contacts at flanking minor grooves facing the protein (fig. S5B). Similar to a bending mode found in the nucleosome (*27*), positive roll, negative slide, and reduced twist are seen in the vicinity of the bend. In addition, but in contrast to known nucleosome positioning sequences such as the 601 sequence, three A-tracts occur in the region with narrowed minor grooves. A-tracts are intrinsically stiff (*28*) and likely influence the curvature of the DNA here. All interactions at this contact site are protein-phosphate contacts. Given the unique DNA shape observed, we posit that shape readout is a major recognition mechanism by UAF at this part of the 35*S* rRNA promoter.

At the Rrn9 contact site, spanning positions −65 to −54, a helix-turn-helix–like element runs parallel to the DNA, making contacts with the DNA backbone at both major and minor grooves and donating one base-specific hydrogen bond at position −57 (Fig. 2A). Arg^295^, in particular, interacts with the sugar moiety of adenine −65 while reaching into a minor groove. This minor groove is compressed, most notably at position −63/−62, which coincides with a TA dinucleotide (fig. S5A). In an adjacent major groove, Lys^308^ interacts with the C2 carbonyl of guanine −57. A separate helix N terminal to the Rrn9 histone fold makes additional contacts with the DNA backbone at positions −55 and −54. Overall, the base-pair step signatures of the Rrn9 contact site do not resemble those of the nucleosome (*27*), suggesting that DNA here is distorted differently. The participation of non–histone-like elements has likely created a different binding surface with distinctly different DNA shape preferences. Combined, the two contact sites of UAF bury 1673 Å^2^ of solvent-accessible DNA surface, spanning 33 bp of DNA. A total of 3346-Å^2^ solvent-accessible surface area is buried in the complex. Given the paucity of base-specific contacts, specificity for DNA appears to be generated from the sum of elements based around the UAF histone core, with shape readout being a major DNA recognition mechanism. In turn, cognate DNA sequence is marked by the presence of stiff A-tracts and an aperiodic distribution of flexible TA dinucleotides, which enable a particular shape.

In contrast to the histone folds of TFIID, the decorated histone-like hexamer of UAF appears to be a main driver of specificity (fig. S6A). In the engaged state of TFIID, the histone-like octamer of lobe A is placed onto DNA by the highly specific binding of the TAF1 winged helix domain and TAF2 to DNA downstream (*11*, *21*, *29*). At the lobe A octamer, only one loop and one helix of three subunits come in contract with DNA, thus constituting a limited interface and likely contributing little to TFIID specificity. Hence, despite both containing histone folds and despite both a catalyst of PIC formation, TFIID and UAF interact with promoter DNA fundamentally differently. In comparison to other histone fold–containing transcriptional regulators, the decorated histone-like hexamer of UAF appears sufficient for generating specificity, independently of cofactors. By contrast, NC2, a H2A/H2B-like regulator of the Pol II PIC assembly process, recognizes DNA that is bent, for example, by TBP (figs. S3A and S6B) (*30*).

### In vitro footprinting identifies UAF and TBP binding sites in the 35*S* rRNA promoter

**Fig. 3.**
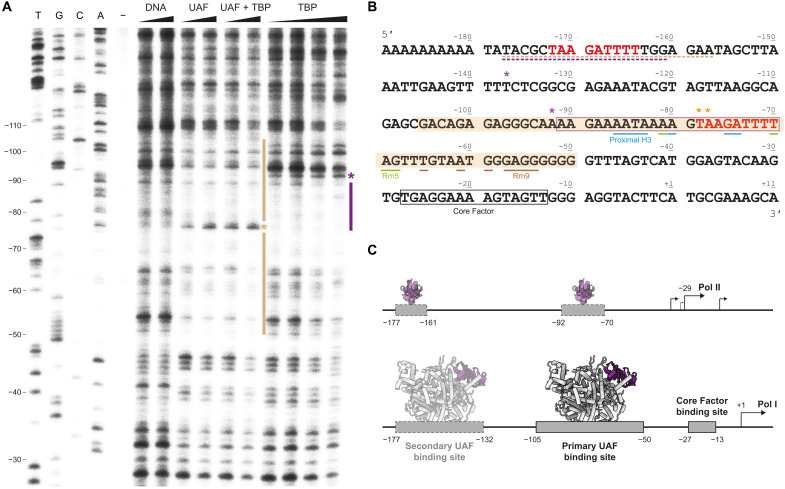
In vitro DNase I footprint of UAF on promoter DNA. (**A**) DNase I footprint visualized by primer extension using the nontemplate strand from position +25 of the 35*S* rRNA promoter. From left to right: Sanger sequencing reactions (T, G, C, and A), untreated DNA (−), free DNA treated with 0.36 and 0.72 U of DNase I (DNA), DNA bound to UAF (4 and 8 μM), UAF and TBP (4 and 8 μM, 1:1 ratio), and TBP (4, 8, 16, and 32 μM), treated with 0.72 U of DNase I. Sequencing lanes are labeled according to the nontemplate strand. Bars indicate protection. Asterisks indicate an increase in sensitivity to DNase I. (**B**) Annotated 35*S* rRNA promoter sequence. The nontemplate strand sequence is displayed. Shaded region indicates the primary UAF binding site. Boxed regions indicate a potential TBP binding site as suggested by the footprint (purple) and known Core Factor binding site (*14*) (black). Potential upstream UAF and TBP binding sites identified in fig. S7A are underlined. Bases contacted by UAF in the structure are indicated. Colored in red is the putative repeat sequence bound by Rrn5 and the proximal H3. (**C**) A schematic model of UAF and TBP binding at the 35*S* rRNA gene promoter. In the absence of UAF, TBP binding to DNA enables Pol II–mediated transcription from upstream of the Pol I transcription start site (*2*, *55*). In the presence of UAF, UAF binding is favored; TBP is sequestered from DNA to support Pol I recruitment.

To dissect the interplay between UAF and TBP on promoter DNA, we conducted a deoxyribonuclease (DNase) I footprinting analysis to examine their individual and collective DNA binding behaviors. In the presence of UAF, we observed protection of DNA from positions −105 to −50 from DNase I, consistent with the protein-DNA interface observed by cryo-EM. A hypersensitive site occurred at position −78/−77, consistent with the bend observed in this region. In the absence of UAF, TBP at equivalent concentrations (4 and 8 μM) gave rise to protection of DNA from positions −92 to −70 (Fig. 3, A and B). Band intensities in this region were more diminished compared with the rest of the lane relative to the DNase I control, suggesting that TBP has a preference for this site in the in vitro setting. At higher concentrations (16 and 32 μM), DNA from −70 to −41 appeared also protected. However, this was accompanied by a reduction in overall lane intensity relative to the control. Considering that TBP can bind DNA nonspecifically at micromolar concentrations (*31*), it is unclear whether TBP also has a preference for this region. By filter binding, we found that TBP in fact binds DNA of the regions −110 to −40 with micromolar or weaker affinity (fig. S7B). By contrast, UAF has 18- to 23-nM affinity for the same region. This marked difference in affinity suggests that although TBP may have preferred binding sites within the 35*S* rRNA gene promoter, the promoter is preferentially bound at the UAF binding site by UAF when both UAF and TBP are present. This is supported by the footprint observed when both factors are present, which appears identical to that of UAF alone, and is consistent with the structure we resolved by cryo-EM.

While in the cryo-EM structure, we did not resolve any protein-DNA contact beyond position −100, the upstream region of −208 to −155 has been reported to contribute weakly to UAF activity in vitro (*6*). Thus, we performed a second footprinting analysis focusing on this region (fig. S7A). Although weak, protection from DNase I was observed from positions −177 to −157 in the presence of UAF and TBP, and UAF alone, indicating a potential secondary UAF binding site here. Comparing between upstream and downstream regions, we found by filter binding that UAF has ~5-fold lower affinity for DNA from −180 to −110 than for DNA from −110 to −40 (fig. S7B). Inspection of the sequence reveals a TAAGATTTT repeat that is present in both regions. This repeat is contacted by Rrn5 and the proximal H3 in the structure (Fig. 3B). Competition filter binding assays reveal that DNA from −180 to −110 and from −110 to −40 compete for UAF binding, suggesting that the binding surfaces of UAF for the two regions are not independent (fig. S7C). Consistent with the measured relative affinities between the two DNA segments, more competitor DNA was required to displace DNA from −110 to −40 from UAF. The lack of a hypersensitive site at position −172/−171, however, which may be expected if DNA was similarly bent at the upstream repeat, suggests that the interface between UAF and DNA at the upstream repeat is not identical. Sequence differences around the repeat may prevent DNA from adopting the shape as seen in the structure, potentially limiting contact between protein and DNA and contributing to the lower affinity observed. Yet still, the potential occupancy of UAF at a secondary binding site upstream corroborates with the double-band pattern previously observed in chromatin endogenous cleavage assays (*7*, *8*) and upstream tail observed in chromatin immunoprecipitation (ChIP)–exo experiments (*9*) and agrees with aforementioned reports of UAF activity in this region (*6*).

### UAF positions TBP for selective Pol I recruitment

**Fig. 4.**
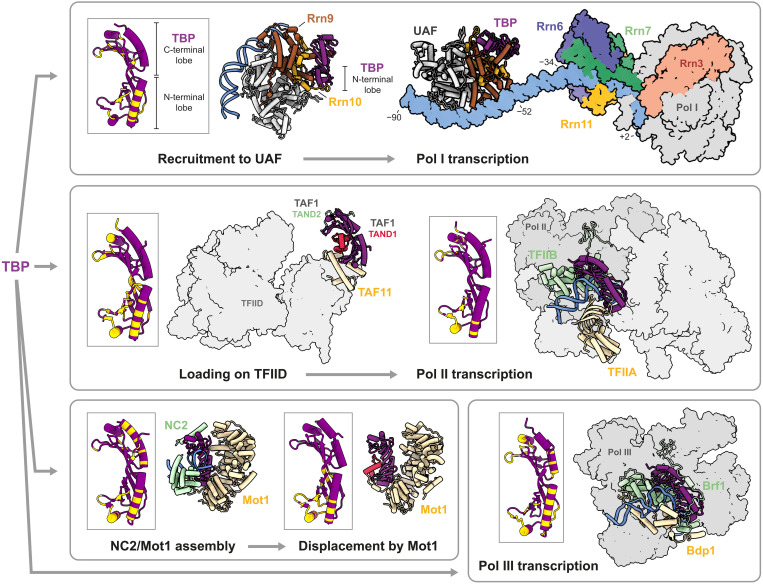
UAF modulates transcription fate through TBP. Top left: TBP is recruited to UAF via UAF subunits Rrn9 and Rrn10 and sequestered from DNA and Pol II/III transcription factors. Inset shows the TBP residues involved in the interface with UAF in yellow. Top right: Structure of UAF-TBP-DNA docked with the minimal Pol I PIC (*14*), comprising Core Factor (Rrn6, Rrn7, and Rrn11), initiation factor Rrn3, Pol I, and DNA (PDB, 6RQL). Middle: TBP is loaded onto TFIID (PDB, 6MZL) (*21*) for subsequent handoff to DNA, TFIIA, and TFIIB for Pol II PIC assembly (PDB, 7O72) (*56*). Bottom left: PIC assembly is blocked by the binding of NC2 and chromatin remodeler Mot1 to promoter-bound TBP (PDB, 4WZS) (*23*). As a result, transcription becomes inhibited. TBP is subsequently displaced from DNA by ATP-dependent Mot1 activity (PDB, 3OC3) (*33*). Note that NC2 also has stimulatory effects on Pol II–mediated transcription at certain promoters (*57*, *58*). Bottom right: TBP partners with Bdp1 and Brf1 to form the TFIIIB complex and initiates Pol III PIC assembly (PDB, 6F42) (*13*). Insets show TBP residues that are involved at each depicted TBP-protein interface.

On the basis of structural studies of the Pol II and Pol III PICs, the fate of a gene during transcription, whether Pol I, II, or III is recruited, is partially dependent on how TBP is placed with respect to promoter DNA. Our structure shows that UAF sequesters TBP from DNA by interacting with both of its lobes (Fig. 4 and fig. S8). At the N-terminal lobe, Rrn9 and Rrn10 contact a conserved regulatory surface (*32*) that is also contacted by TFIID and TFIIA during Pol II PIC assembly and by Brf1 during Pol III PIC assembly. These Pol II– and Pol III–specific transcription factors are thus prevented from interacting with TBP at the 35*S* rRNA promoter. In addition, the N-terminal helix of Rrn9 engages in hydrophobic interactions with the DNA binding surface of TBP. These interactions are reminiscent of those at the interface of TFIID subunit TAF1 with TBP and chromatin remodeller Mot1 with TBP (fig. S9). In the canonical state of TFIID, TBP is momentarily inhibited from binding DNA before it is handed off to TFIIA and DNA. In the case of Mot1, concerted steric exclusion of TFIIA and TFIIB, together with NC2, facilitates the displacement of TBP from transcriptionally active genes (*23*, *33*). We speculate that TBP bound to UAF is prevented from searching the 35*S* rRNA gene promoter for high-affinity binding sites. By simultaneous occlusion of the TFIID, TFIIA, and Brf1 binding site, UAF acts to inhibit Pol II and Pol III PIC formation, thus establishing Pol I as the preferred polymerase for 35*S* rRNA transcription. Deletion of Rrn9 or Rrn10, which on the basis of the structure would impair DNA binding or TBP sequestration, leads to increased chromatin accessibility by TBP and a switch to rRNA transcription by Pol II (*2*–*5*).

It remains an open question how UAF participates in the Pol I PIC. In the present structure, all TBP residues found to cross-link with Core Factor (*34*), except Lys^110^ and Lys^138^, are solvent accessible. The structure of TBP in association with UAF is therefore permissive to higher-order assembly with Core Factor. Docking of the UAF-TBP-DNA structure with structures of the minimal PIC comprising Core Factor, initiation factor Rrn3, Pol I, and downstream DNA (*14*–*16*) suggests that UAF and TBP are positioned 18 bp away from Core Factor at the 35*S* rRNA promoter (Fig. 4). For TBP to contact Core Factor and act as an adaptor between UAF and Core Factor as hypothesized (*10*), bending of the DNA between or a conformational rearrangement may be required. It is yet unclear whether UAF and Core Factor interact directly with each other in the complete Pol I PIC assembly. Nevertheless, it is evident from the structural and biochemical analyses here that UAF plays a crucial role in Pol I selection during transcription initiation.

## DISCUSSION

Here, we have focused on the molecular mechanisms of Pol I selection by budding yeast UAF. By cryo-EM, we found that UAF contains a hexameric histone-like core, composed of subunits Rrn5, two histone H3, one H4, Rrn9, and Rrn10 (Fig. 1). Subunit Rrn5, one H3, and Rrn9 make multiple contacts with the 35*S* rRNA gene promoter from positions −86 to −54 (Fig. 2). A common feature of transcription factor complexes (*35*), the multiple contacts combine to drive strong and specific binding at the promoter with ~20 nM affinity, as determined by DNase I footprinting and filter binding assays (Fig. 3 and fig. S7). The abundance of DNA backbone contacts observed in the cryo-EM structure and the distinct shape of the bound DNA indicate that specificity is generated through shape recognition. In turn, the cognate DNA sequence is marked by an aperiodic distribution of TA dinucleotide steps and the presence of 4-nucleotide-long A-tracts. Last, by positioning TBP away from DNA and such that TFIIA, TFIIB, TFIID, Mot1, NC2, and Brf1 but not Core Factor are sterically excluded from binding TBP, UAF favors Core Factor recruitment and thereafter Rrn3 and Pol I recruitment to the 35*S* rRNA gene promoter (Fig. 4).

Beyond its essential role in Pol I recruitment, UAF has been implicated in the structural organization of the nucleolus. Deletion of UAF subunits not only causes a switch to Pol II rRNA transcription but also causes detachment of the crescent-shaped nucleolus from the nuclear periphery (*3*). In particular, deletion of Uaf30 leads to reduced levels of high mobility group protein Hmo1 and the Pol II–silencing histone deacetylase Sir2 at the rRNA promoter (*5*). Given its location within the UAF complex and repeated observations that Uaf30 phosphorylation correlates with Pol I activity (*36*–*38*), Uaf30 is potentially a phosphorylation-dependent interaction hub key to the maintenance of a Pol I–selective, nucleolus-specific chromatin architecture. In budding yeast, rRNA genes are clustered in series on the same chromosome. Repeated DNA bending at UAF- and Core Factor–bound promoters over consecutive active genes may further contribute to a nucleolus-specific chromatin architecture through torsional and steric effects.

Although the 35*S* rRNA gene promoter is a TATA-less promoter, TATA-like elements, sequences that have fewer than two mismatches to the TATA box consensus sequence TATAWAWR (*39*), can be found throughout the promoter at positions −192, −130, −120, −88 to −77, and −35. TATA-like elements have been strongly correlated with TBP and general transcription factor occupancy in the TATA-less promoters of Pol II–transcribed genes. In our in vitro analysis, we detected a weak footprint by TBP from positions −92 to −70, which coincides with a series of overlapping TATA-like elements. These elements are likely to be occluded in the presence of UAF because of its higher affinity for the region. Adjacent elements are likely also occluded because of secondary UAF binding upstream and Core Factor binding downstream. However, upon disruption of UAF function, these elements could become available to support Pol II PIC assembly. Thus, it would seem that yeast rRNA transcription is the result of opposing forces whose balance is tipped in favor of Pol I recruitment by Pol I–specific factors under normal circumstances. It is unknown if in metazoans TBP is positioned similarly in the Pol I PIC. However, an intricate interplay between transcription factors likely also exists and underlies the recruitment of Pol I.

From a protein evolutionary perspective, the structure of UAF underscores the adaptability of the histone fold as a scaffold for specific DNA recognition. While the fold features also in TFIID, SAGA, and NC2, the histone folds of UAF have been co-opted to recognize a specific and continuous 33-bp DNA element independently of cofactors. Together with the recent discovery of an RNA motif–specific H2A-H2B dimer in human telomerase (*40*), it becomes increasingly apparent that the histone fold is a versatile platform that can be harnessed to target specific nucleic acid elements for function, beyond shaping nucleic acids nonspecifically or mediating protein-protein interactions. Here, we show how the histone fold is harnessed to suppress Pol II recruitment in support of Pol I PIC assembly at a specific gene promoter. Given the broader role of UAF in shaping nuclear architecture, our findings provide a framework for further dissection of the mechanisms involved.

## MATERIALS AND METHODS

### Production of UAF

UAF was produced recombinantly in *E. coli* by coexpression of all subunits from three plasmids: pRSFDuet-1 (Novagen) encoding a His_6_-TEV-SUMO-Rrn9 fusion and Rrn10, pCDFDuet-1 (Novagen) encoding Uaf30 and a His_6_-TEV-SUMO-Rrn5 fusion, and pETDuet-1 (Novagen) encoding histones H3 and H4. Coding sequences for Rrn5, Rrn9, Rrn10, and Uaf30 were codon optimized, synthesized (Genscript), and subcloned. Autoinduction was carried out in LOBSTR-BL21(DE3) cells (Kerafast) in ZYP-5052 medium where ZY was substituted with 1.2% (w/v) tryptone and 2.4% (w/v) yeast extract. Medium supplemented with kanamycin, streptomycin, and ampicillin was inoculated at 37°C and grown to an OD_600nm_ (optical density at 600 nm) of 0.9. The temperature was then reduced to 18°C, and the culture was grown for 16 hours further. Cells were harvested by centrifugation and lysed with a M-110L microfluidizer processor (Microfluidics) in 20 mM tris-HCl (pH 7.5), 400 mM ammonium sulfate, 20% (v/v) glycerol, 20 mM imidazole, and 10 mM β-mercaptoethanol, with 1× cOmplete protease inhibitor cocktail (Roche). Supernatant after centrifugation was incubated with Ni–nitrilotriacetic acid (NTA) agarose (Qiagen) beads for 1 hour at 4°C. Beads were washed twice with 20 mM tris-HCl (pH 7.5), 500 mM KCl, 20 mM imidazole, 20% (v/v) glycerol, and 10 mM β-mercaptoethanol. Protein was eluted in the same buffer supplemented with 300 mM imidazole. Eluate was dialyzed overnight at 4°C with SUMO protease [EMBL PepCore Facility, 1:100 (w/w)] against 20 mM tris-HCl (pH 7.5), 400 mM KCl, 20% (v/v) glycerol, 10 mM imidazole, and 10 mM β-mercaptoethanol. The protein was further purified on a Mono S 10/100 GL column (GE Healthcare) using a 400 to 1000 mM KCl gradient in 20 mM tris-HCl (pH 7.5), 10% (v/v) glycerol, and 10 mM β-mercaptoethanol, followed by size exclusion chromatography on a Superdex 200 10/300 GL column (GE Healthcare) in 20 mM Hepes-NaOH (pH 7.5), 300 mM KCl, 5% (v/v) glycerol, and 2 mM dithiothreitol (DTT). Fractions corresponding to UAF as determined by SDS–polyacrylamide gel electrophoresis analysis were concentrated to 10 mg/ml on an Amicon centrifugal concentrator (Millipore), molecular weight cutoff of 3000 Da, at 4°C and flash frozen for storage at −80°C.

### Production of TBP

Full-length TBP with an N-terminal His_6_-tag and TEV cleavage site was expressed from a pET-MCN-EAVNH vector in Rosetta 2 pLysS *E. coli* strain using a TB medium at 37°C supplemented with ampicillin and chloramphenicol. When OD_600nm_ reached 0.9, 400 μM isopropyl-β-d-thiogalactopyranoside (IPTG) was added to the medium and the culture was grown for 4 hours. Cells were harvested by centrifugation and lysed with a M-110L Microfluidizer Processor in 50 mM tris-HCl (pH 7.6), 200 mM KCl, 20% (v/v) glycerol, 5 mM imidazole, 5 mM MgCl_2_, 10 mM β-mercaptoethanol, and protease inhibitor cocktail (Roche). Following centrifugation, the supernatant was incubated with Ni-NTA Agarose (Qiagen) beads for 1 hour at 4°C. Beads were washed with buffer containing 20 mM tris-HCl (pH 7.6), 500 mM KCl, 20% (v/v) glycerol, 5 mM imidazole, and 10 mM β-mercaptoethanol. Protein was eluted in 20 mM tris-HCl (pH 7.6), 100 mM KCl, 20% (v/v) glycerol, 250 mM imidazole, and 10 mM β-mercaptoethanol. The protein was diluted in 20 mM tris-HCl (pH 7.8), 200 mM KCl, 20% (v/v) glycerol, 0.1 mM EDTA (pH 8.0), and 1 mM DTT and further purified on a 5-ml HiTrap Heparin HP column (GE Healthcare), eluting with a 200 to 600 mM KCl gradient. Eluate was dialyzed overnight at 4°C with TEV protease [EMBL PepCore Facility, 1:100 (w/w)] against 20 mM tris-HCl (pH 7.8), 100 mM KCl, 20% (v/v) glycerol, 20 mM imidazole, and 1 mM DTT. The protein was further purified on a Mono S 10/100 GL column (GE Healthcare) using a 100 to 400 mM KCl gradient in 20 mM tris-HCl (pH 7.8), 20% (v/v) glycerol, 0.1 mM EDTA, and 1 mM DTT, followed by size exclusion chromatography on a Superdex 75 10/300 GL column (GE Healthcare) in 20 mM Hepes-NaOH (pH 7.5), 200 mM KCl, 20% (v/v) glycerol, 5 mM magnesium acetate, and 1 mM DTT. Fractions corresponding to TBP were concentrated to 20 mg/ml on an Amicon centrifugal concentrator (Millipore), molecular weight cutoff of 3000 Da, at 4°C and flash frozen for storage at −80°C.

### Reconstitution of UAF and TBP on promoter DNA

DNA substrate for cryo-EM spanning positions −190 to −40 was prepared by PCR using the plasmid pNOY378 (*6*) as template. The plasmid contains a 35*S* rRNA gene fragment from positions −221 to +951 relative to the transcription start site. Oligonucleotides 5′-GAAAAAAAAAATATACGCTAAGATTTTTGG-3′ and 5′-ATGACTAAACCCCCCCTCC-3′ synthesized and high-performance liquid chromatography (HPLC) purified by Sigma-Aldrich were used. The reaction was performed using Phusion polymerase (Thermo Fisher Scientific), an annealing temperature of 63°C, annealing time of 15 s, and an extension time of 10 s. The PCR product was purified on a MonoQ 10/100 column (GE Healthcare) with a 0.5 to 1 M NaCl gradient in 20 mM tris-HCl (pH 7.5), 1 mM EDTA (pH 8.0), over 50 column volumes. Fractions were pooled and further purified by phenol-chloroform extraction and ethanol precipitation and, lastly, resuspended in water. To reconstitute the UAF-TBP-DNA complex for cryo-EM imaging, UAF at 53 μM stock concentration was mixed with TBP at 140 μM at a 1:2 molar ratio and incubated on ice for 10 min. An equimolar amount of the mixture was added to DNA in reconstitution buffer [30 mM Hepes-NaOH (pH 7.5), 30 mM sodium citrate (pH 6.0), 250 mM KCl, and 2 mM DTT] to a final concentration of 3 μM and incubated for 20 min on ice. To prepare UAF-TBP, UAF and TBP were mixed 1:2 as above and diluted in 20 mM sodium citrate (pH 6.0), 250 mM KCl, and 2 mM DTT.

### Cryo-EM imaging and structure determination

Freshly assembled complexes were mixed 1:0.92 (v/v) with 0.1% (w/v) lauryl maltose neopentyl glycol (Anatrace) in reconstitution buffer and applied to UltrAuFoil R1.2/1.3 grids, 300 mesh, glow discharged in a PELCO easiGlow system, and plunge frozen at 100% humidity and 10°C into liquid ethane in a Vitrobot Mark IV (Thermo Fisher Scientific). Grids were screened on a 200-kV Talos Arctica (Thermo Fisher Scientific). Data for UAF-TBP-DNA were collected over two sessions and two separate grids on a 300-kV Titan Krios G3 (Thermo Fisher Scientific) equipped with a Gatan K3 detector and energy filter using SerialEM (*41*) at a pixel size of 0.645 Å/pixel, total electron dose of 49.4 to 49.6 e^−^/Å^2^ over 40 frames, defocus of −0.9 to −1.9 μm, and a slit width of 20 eV. Data for UAF-TBP were collected with the same imaging parameters from one grid in one microscope session. Particles were picked in cryoSPARC (*42*) with the blob picker, allowing up to 360-Å diameter. For UAF-TBP-DNA, particles from the two datasets were pooled. Following two-dimensional (2D) classification, 1,479,233 particles were selected and an ab initio model was generated using cryoSPARC. Particle coordinates from the 2D classification were subsequently used for particle extraction in RELION v3.1.2 (*43*) with a binning factor of 4. 3D classification yielded one class with well-defined densities for all components of UAF except Uaf30 (fig. S1C). This class containing 193,226 particles was chosen for further refinement, Bayesian polishing, contrast transfer function (CTF), and aberration refinement, which lastly yielded a map at 2.8-Å resolution by gold standard Fourier shell correlation (FSC). To better visualize the upstream end of the assembled complex, masked classification was performed, which produced one class with density connecting DNA to the Uaf30 region. For model building in Coot (*44*), the 2.8-Å-resolution map was postprocessed using DeepEMhancer (*45*). Available structures of budding yeast histones (*26*) and TBP (*46*) were placed into their respective densities, whereas subunits Rrn5, Rrn9, Rrn10, and Uaf30 were built de novo, since no homologous structures were available. B-DNA was fitted with self-restraints into the density observed. Sugar and base bond angle and length restraints were generated using RestraintLib (*47*, *48*) for real space refinement in Phenix (*49*). Real space refinement was performed with additional secondary structure restraints for protein and no noncrystallographic symmetry constraints between the two copies of histone H3 in UAF against the unsharpened consensus map from 3D refinement. The refined structure was validated with MolProbity (*50*). DNA geometry was analyzed with Curves+ (*51*). Structures were visualized using ChimeraX (*52*). Electrostatic potential calculations were performed using APBS and PDB2PQR (*53*) under the PARSE force field. Buried surface area calculations were performed using PISA (*54*).

### DNase I footprinting

The plasmid pNOY378 (*6*) was linearized with Bgl II, which produces a single cut at position +124. DNA (1 μM) was incubated with UAF (4 and 8 μM), TBP (4, 8, 16, and 32 μM), or a mixture of UAF and TBP at 1:1 ratio (4 and 8 μM), in reconstitution buffer for 15 min on ice. Next, the reaction was supplemented with 2.5 mM MgCl_2_ and 0.5 mM CaCl_2_, and DNase I (New England Biolabs) was added to a final concentration of 0.075 U/μl and the reaction incubated for 5 min at 28°C. Last, DNA was purified by phenol-chloroform extraction, followed by ethanol precipitation, and resuspended in water. As controls, DNase was omitted in one reaction, and protein in another. To visualize the cleavage pattern, primer extension was performed using the DNA cycle sequencing kit (Jena Bioscience), 150 ng of digested DNA, and ^32^P-end–labeled oligonucleotides according to the manufacturer’s protocol. An annealing temperature of 55°C and 25 thermal cycles were used. The oligonucleotides used were 5′-AACTTGTCTTCAACTGCTTTCGC-3′, corresponding to positions +25 to +3 of the 35*S* rRNA gene, and 5′-CATGGTCGGGCACCTGTC-3′, corresponding to positions −214 to −197. Sanger sequencing reactions were performed alongside using undigested DNA by supplementing dideoxynucleotide triphosphates (ddNTPs) at 10 μM final concentration. After addition of 4 μl of loading buffer [95% (v/v) formamide, 1× TBE, 0.025% (w/v) xylen cyanol, bromphenol blue], reactions were heated for 3 min at 95°C and analyzed on a denaturing 8% (w/v) polyacrylamide gel (19:1 acrylamide-bisacrylamide, 8.3 M urea, 1× TBE). The gel was exposed to a phosphorimaging screen (Fujifilm), which was then scanned using a Typhoon FLA 9500 laser scanner (GE Healthcare).

### Filter binding and competition assays

Oligonucleotides (Sigma-Aldrich, HPLC purified) corresponding to both strands of the 35*S* rRNA promoter from positions −180 to −110 and −110 to −40 were end labeled using [γ-^32^P] adenosine 5′-triphosphate (ATP) and T4 polynucleotide kinase (New England Biolabs) and purified on a 10% acryl/bisacrylamide, 8.3 M (w/v) urea gel. DNA was eluted overnight from excised gel bands in 0.5 M ammonium acetate, 10 mM magnesium acetate, 0.1% (w/v) SDS, 0.1 mM EDTA, and then ethanol precipitated. Last, complementary, labeled strands were annealed at room temperature in 20 mM Hepes-NaOH (pH 7.5), 5 mM MgCl_2_, 100 mM KCl for 30 min. Filter binding assays were performed as follows. DNA (~30,000 cpm, ~10 nM) was incubated with increasing amounts of UAF (0.5 nM to 5 μM) in reconstitution buffer for 1 hour at 4°C and then filtered through a 0.45-μm nitrocellulose filter (Whatman). Filters were counted in a Tri-Carb 2800TR Cerenkov scintillation counter (Perkin Elmer). Counts were normalized, and a Hill equation with a fixed Hill coefficient of 1 was fitted using Prism (GraphPad). Competition assays were performed at ~60% protein occupancy based on estimated *K*_d_ values. Therefore, 100 nM UAF was mixed with 9000 cpm (~3 nM) radiolabeled upstream DNA and 60 nM UAF with 9000 cpm (~3 nM) radiolabeled downstream DNA. Formed complexes were challenged with unlabeled downstream and upstream DNA (5 nM to 20 μM), respectively. DNA retention on nitrocellulose filters was measured as in above filter binding assays.

## Supplementary Materials

### This PDF file includes:

Figs. S1 to S9 Tables S1 to S2 References
