# Supplementary material for: Mechanism of RNA polymerase I selection by transcription factor UAF
Source: Sci Adv. 2022 Apr 20;8(16):eabn5725. doi: 10.1126/sciadv.abn5725 (PMC9020658; doi:10.1126/sciadv.abn5725)
Supplement: Supplementary file 1 — Figs. S1 to S9 Tables S1 to S2 References [file sciadv.abn5725_sm.pdf]

Science Advances
AAAS

Supplementary Materials for

Mechanism of RNA polymerase I selection by transcription factor UAF

Florence Baudin, Brice Murciano, Herman K. H. Fung, Simon A. Fromm, Simone Mattei, Julia Mahamid, Christoph W. Müller*

*Corresponding author. Email: christoph.mueller@embl.de

Published 20 April 2022, *Sci. Adv.* **8**, eabn5725 (2022)
DOI: 10.1126/sciadv.abn5725

This PDF file includes:

Figs. S1 to S9
Tables S1 to S2
References

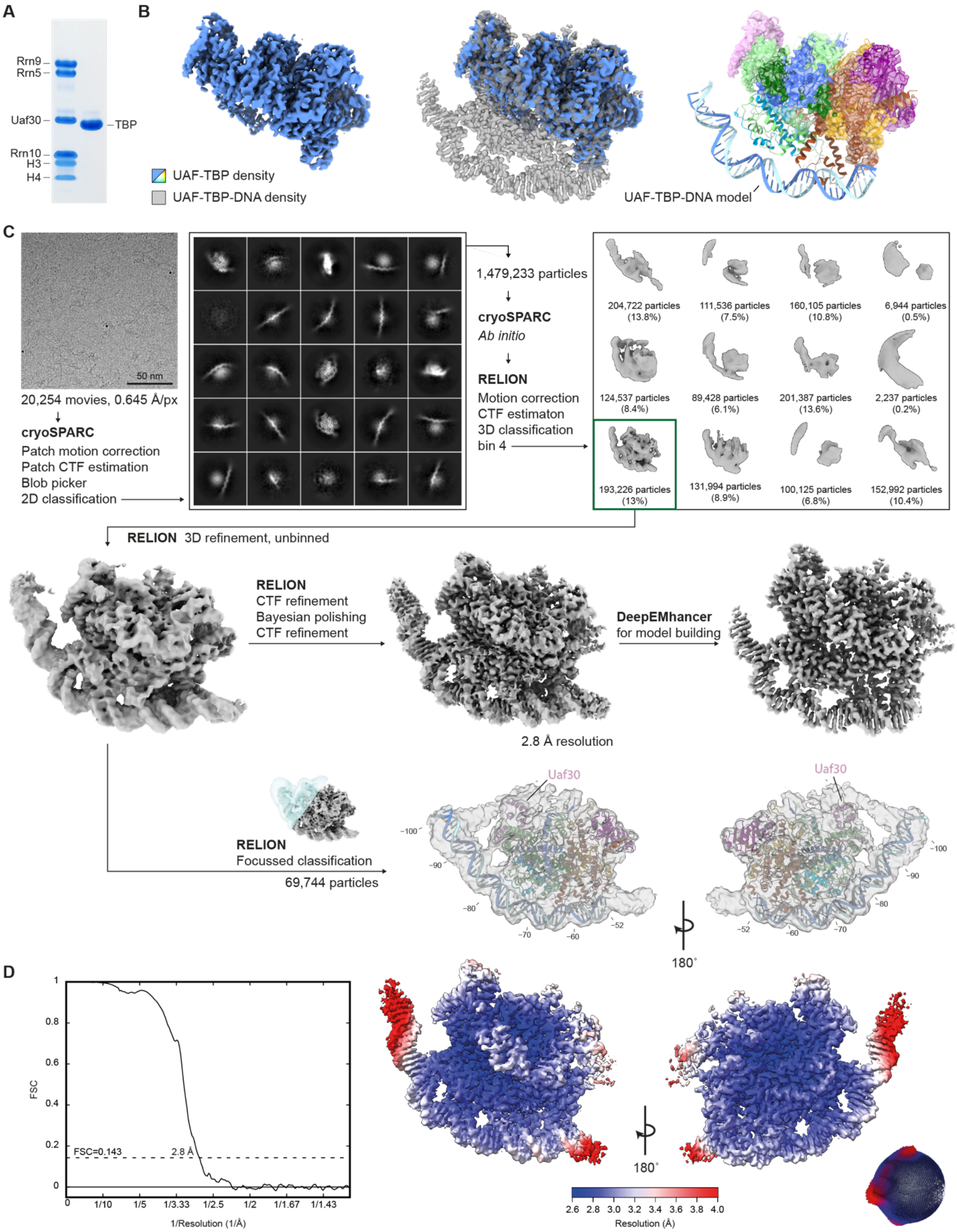
A
Rrn9
Rrn5
Uaf30
Rrn10
H3
H4
TBP
B
UAF-TBP density
UAF-TBP-DNA density
UAF-TBP-DNA model
C
50 nm
20,254 movies, 0.645 Å/px
cryoSPARC
Patch motion correction
Patch CTF estimation
Blob picker
2D classification
1,479,233 particles
cryoSPARC
Ab initio
RELION
Motion correction
CTF estimaton
3D classification
bin 4
204,722 particles (13.8%)
111,536 particles (7.5%)
160,105 particles (10.8%)
6,944 particles (0.5%)
124,537 particles (8.4%)
89,428 particles (6.1%)
201,387 particles (13.6%)
2,237 particles (0.2%)
193,226 particles (13%)
131,994 particles (8.9%)
100,125 particles (6.8%)
152,992 particles (10.4%)
RELION 3D refinement, unbinned
RELION
CTF refinement
Bayesian polishing
CTF refinement
DeepEMhancer
for model building
2.8 Å resolution
RELION
Focussed classification
69,744 particles
Uaf30
180˚
D
FSC
FSC=0.143
2.8 Å
1/Resolution (1/Å)
Resolution (Å)
180˚

Fig. S1.

Cryo-EM data collection and processing. (**A**) Coomassie blue-stained SDS-PAGE gel of purified UAF (left) and TBP (right). (**B**) Cryo-EM density map of UAF-TBP (blue), obtained from 117 598 particles of separately reconstituted and imaged UAF-TBP. Middle, superposition of the UAF-TBP density map (blue) with the UAF-TBP-DNA map (gray). Right, superposition of the UAF-TBP map with the refined model of UAF-TBP-DNA, represented as a cartoon. The UAF-TBP map is coloured by subunit to indicate more clearly which elements are unresolved in the absence of DNA. (**C**) Processing of the UAF-TBP-DNA data. Shown are a representative micrograph, 2D classes from CryoSPARC, 3D classes from RELION, and RELION-post-processed and DeepEMhancer-filtered density maps. The atomic model of UAF-TBP-DNA was built using the DeepEMhancer-filtered map and refined against the unsharpened consensus map. The consensus map following focused classification on the upstream region is shown, low-pass-filtered to 5 Å resolution. The atomic model is docked in and DNA extended to reveal a putative Uaf30 contact site. (**D**) FSC curve of UAF-TBP-DNA (left), local resolution and angular distributions (right) calculated using RELION (*43*).

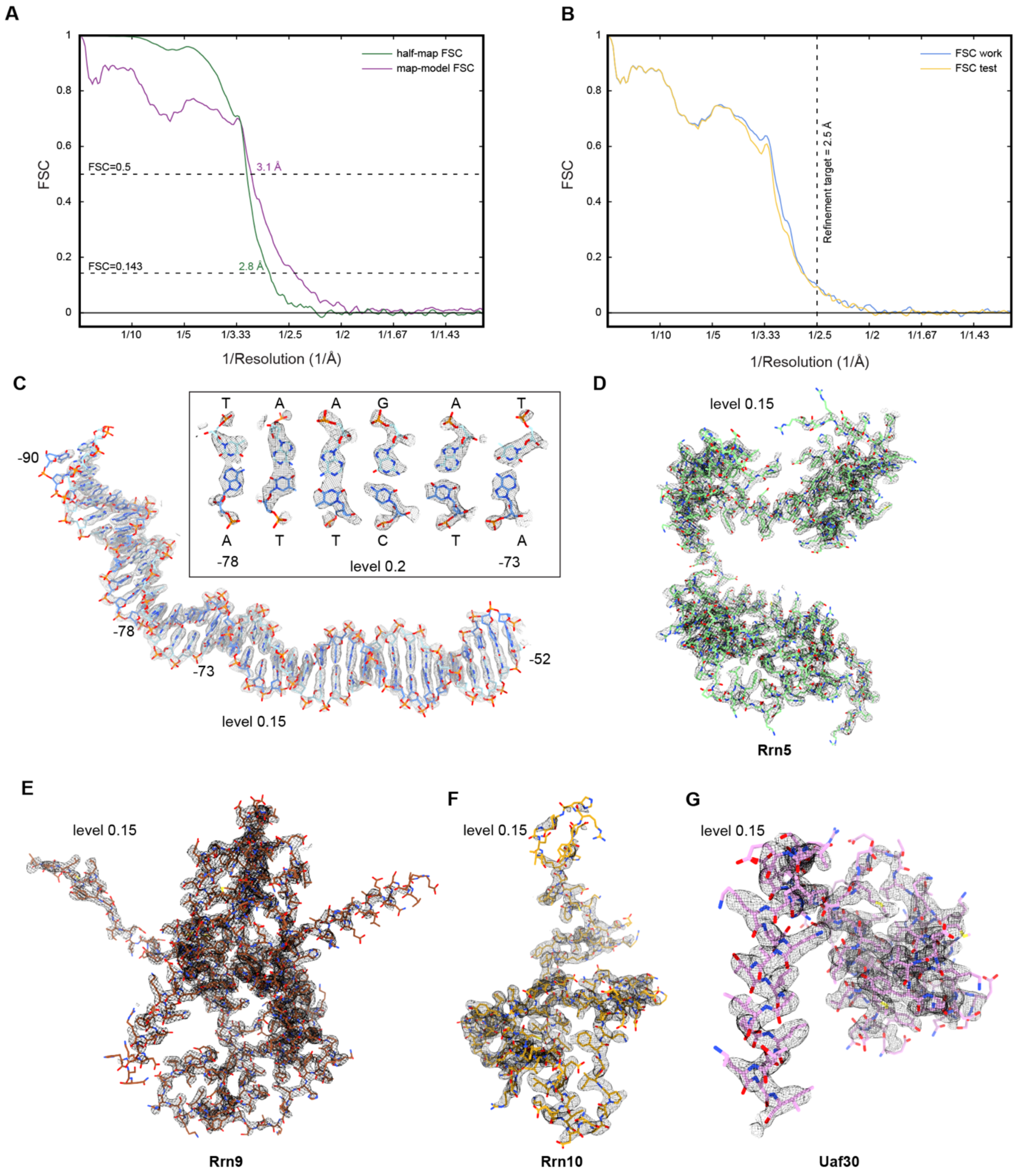

Fig. S2.

Model validation and density fit. (**A**) FSC between half-maps, and between the consensus map and the model to assess map-to-model fit. (**B**) Cross-validation test to assess overfitting during model refinement. FSC between the model refined against half-map A and half-map A (FSC work) or half-map B (FSC test). The resolution target during model refinement is indicated. (**C-G**) Exemplary cryo-EM densities and refined model of promoter DNA, Rrn5, Rrn9, Rrn10 and Uaf30.

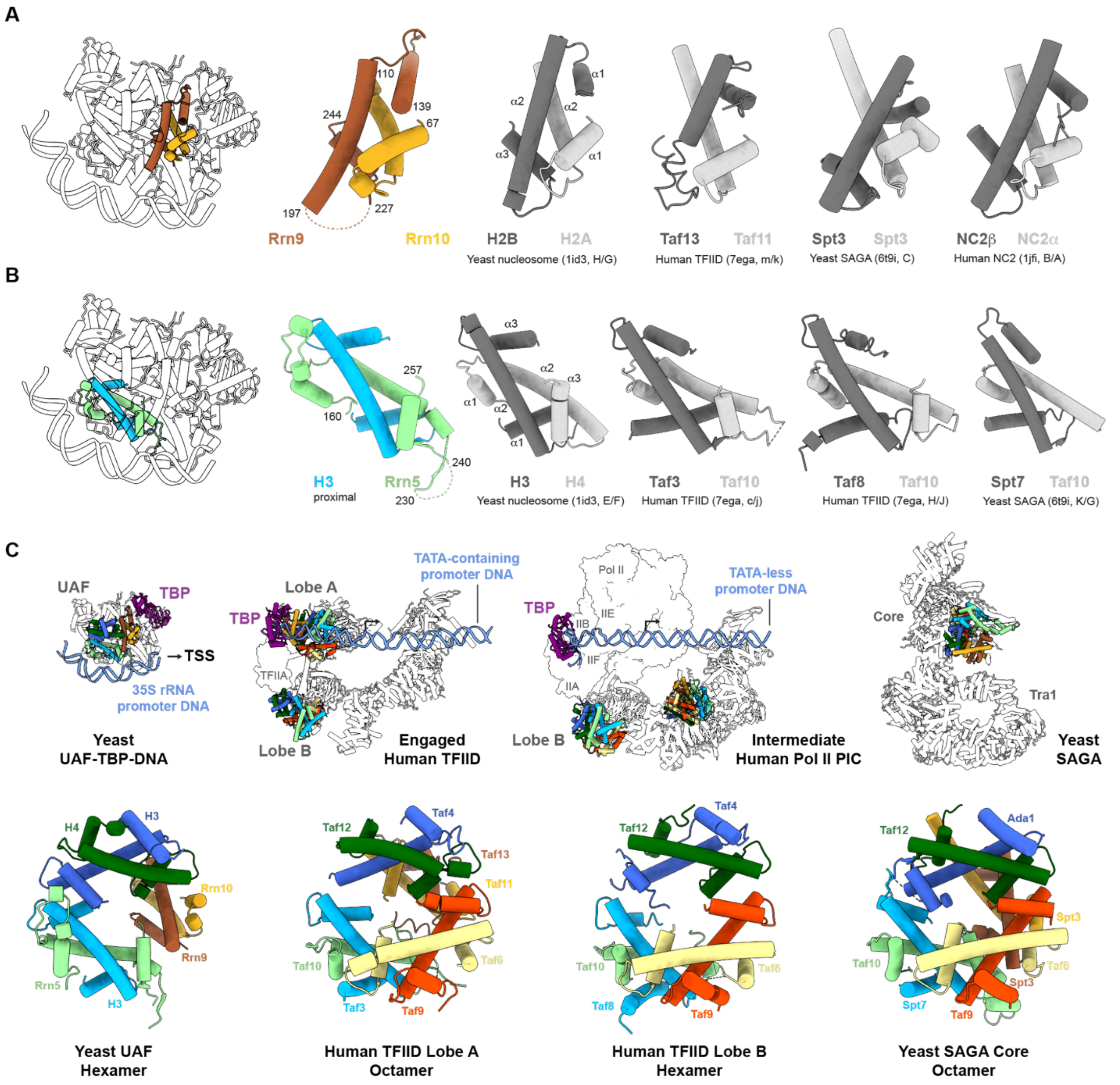

Fig. S3.

Histone fold comparisons. (**A**) The H2B-H2A-like histone fold domains of Rrn9 and Rrn10, and (**B**) H4-like histone fold domain of Rrn5 in complex with H3, juxtaposed with homologs from the yeast nucleosome (PDB 1ID3), human TFIID (PDB 7EGA), yeast SAGA (PDB 6T9I) and human NC2 (PDB 1JFI). (**C**) The histone-like hexamers and octamers of UAF, TFIID and SAGA. Top, the locations of TBP and DNA relative to each histone-like substructure are shown. TFIID is shown in the engaged, initial loading state (PDB 7EGD) and as part of the intermediate PIC (PDB 7EGA), with other proteins represented as surfaces. Bottom, montage of the histone-like hexamers and octamers. Subunits are coloured according to the nucleosome.

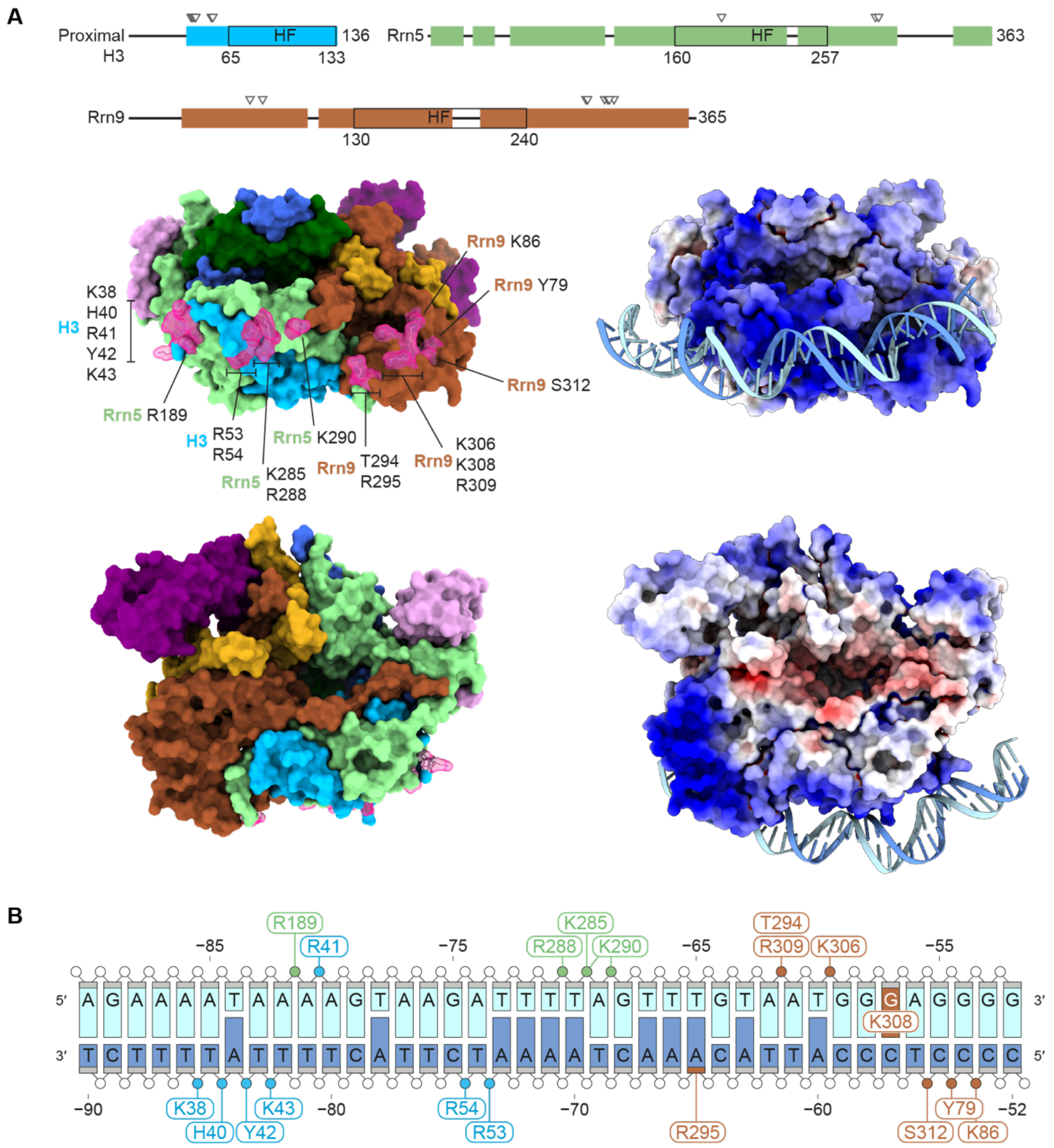

Fig. S4.

UAF-DNA contacts. (**A**) Interacting protein residues are indicated by triangles on the domain diagrams (top) and highlighted in pink on the surface representations of UAF and TBP (left), with DNA hidden. Electrostatic potentials (+10 kT/*e*, blue; −10 kT/*e*, red) are displayed in the same orientation on the right, with DNA displayed as ribbons and sticks. (**B**) Schematic representation of the visualized contacts. Contacted phosphates (circle), sugars (small rectangle) and bases (rungs) are highlighted based on the protein subunit involved and the contacting amino acid residue is indicated.

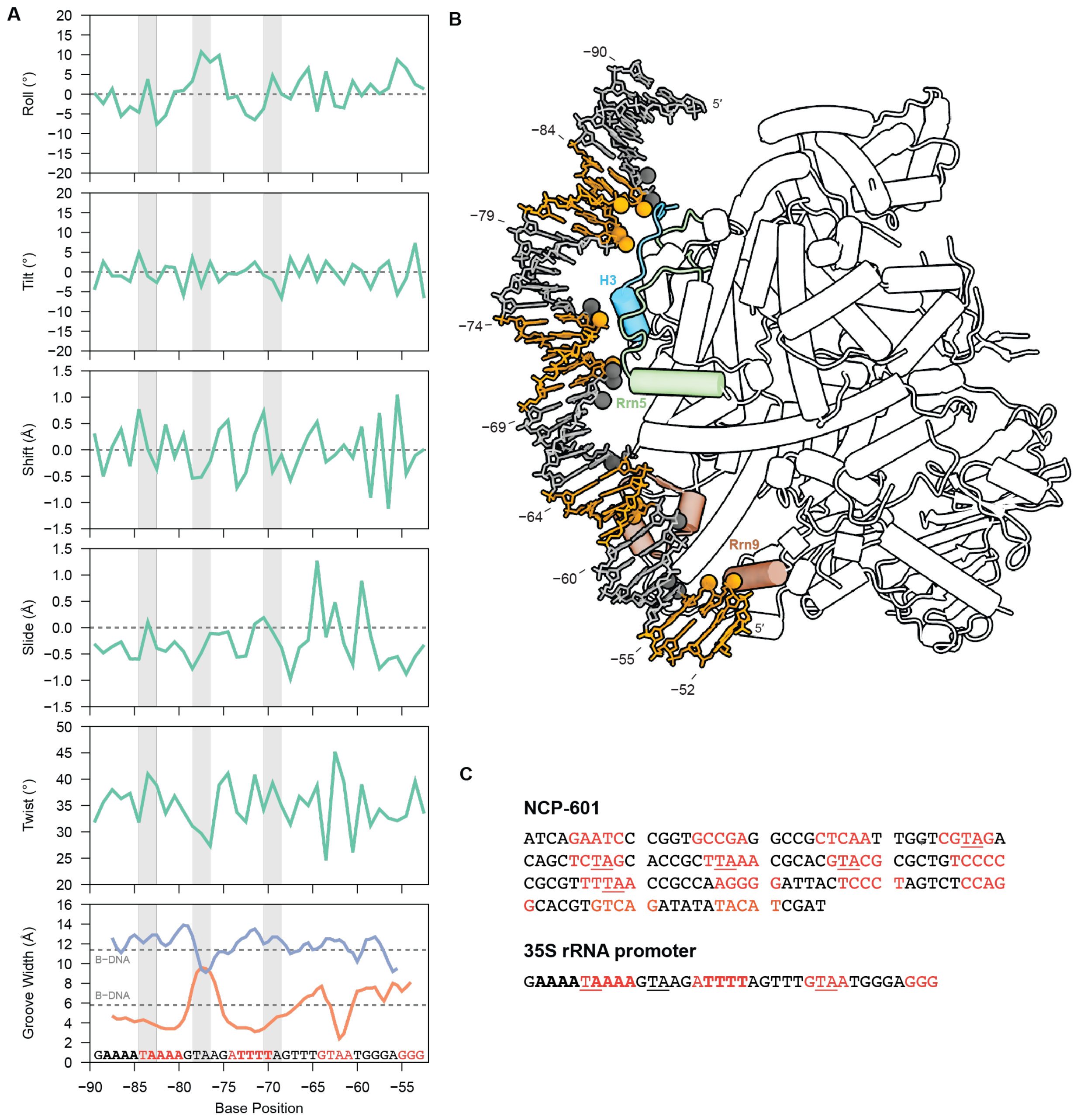

Fig. S5.

UAF-bound DNA is distinctly bent. (**A**) Inter-base-pair parameters of UAF-bound DNA. The translational (shift, slide) and rotational (roll, tilt, twist) parameters of each dinucleotide step resolved in the structure was calculated using Curves+ (*51*) and shown. Major and minor groove widths (spline-based and taking into account the radius of the phosphodiester backbone) are plotted in the bottom, with the non-template strand sequence overlaid underneath. A tracts are displayed in bold and TA dinucleotides are indicated with shaded bars. Bases are coloured according to whether the minor groove (dark orange) or major groove (black) is facing inward towards the protein. (**B**) UAF contact points on DNA. DNA atoms in contact with protein are displayed as spheres. Bases are coloured as in A. Protein secondary structure elements participating at the

interface are coloured by subunit. (**C**) Comparison of UAF-bound DNA with the 601 nucleosome positioning sequence. Bases at an inward-facing minor groove are coloured dark orange; major groove, black, based on Chua et al. (*59*). TA dinucleotides are underlined. A tracts (absent in the 601 sequence) are shown in bold. The central position of the 601 sequence is indicated with an arrow above.

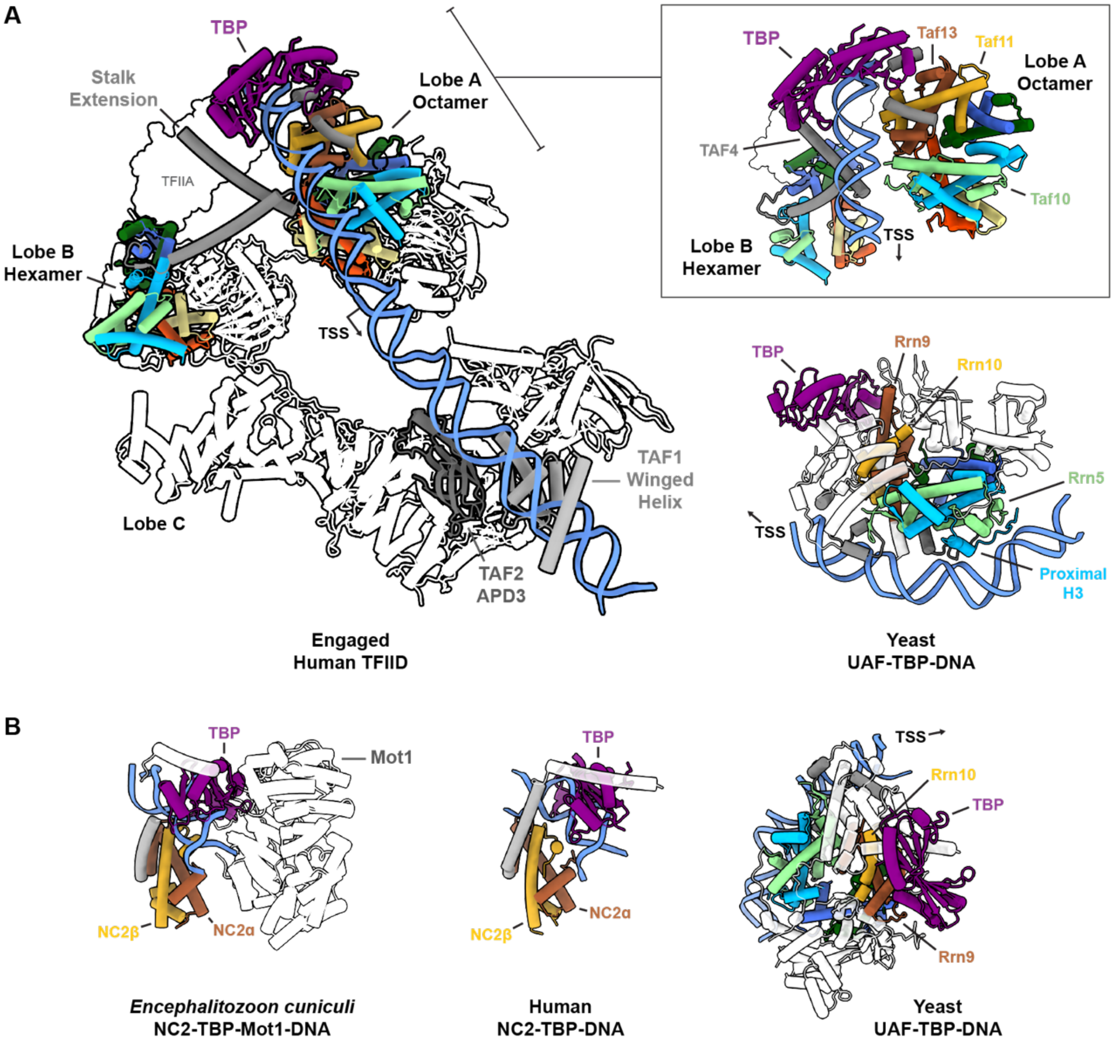

Fig. S6.

UAF binds DNA differently from the histone-fold-containing TFIID and NC2. (**A**) Comparison of DNA-binding elements in TFIID and UAF. The histone-like octamers and hexamers of each complex are coloured according to the nucleosome. Non-histone-like DNA-binding elements are coloured in gray. For TFIID (PDB 7EGD), this includes the TAF1 winged helix domain and TAF2 aminopeptidase-like domain 3 (APD3) of lobe C, and the stalk extension of TAF4 of the lobe B histone-like hexamer. Insert shows the upstream TFIID-DNA contact site in a different orientation, aligned based on TAF3 of the lobe A octamer (light blue) with the proximal H3 of UAF (bottom). Notably, DNA is bound in a different orientation with respect to the histone-like scaffold. Contract of the lobe A octamer with DNA (via TAF10, TAF11 and TAF13) is relatively limited compared to the UAF-DNA interface. (**B**) Comparison of DNA-binding elements in NC2 and UAF. Structures are aligned based on their respective H2A/H2B-like subunits (yellow and orange). Histone-like elements and non-histone-like DNA-binding elements are coloured according to the nucleosome and gray, respectively. The H2A-H2B-like NC2 dimer accesses DNA that is bound and bent by TBP from the opposite side of TBP (PDB 4WZS, 1JFI). The H2B- and H2A-like histone folds of Rrn9 and Rrn10 do not engage DNA.

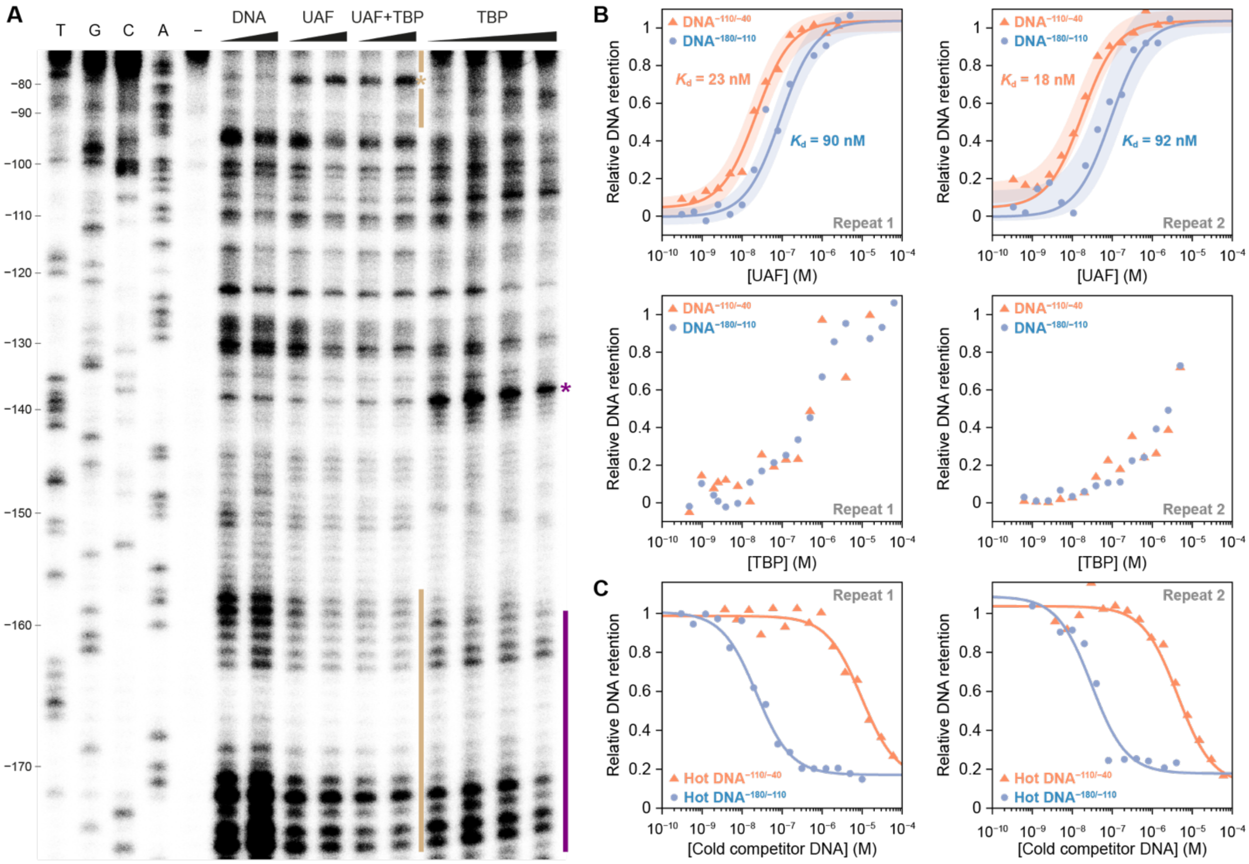

Fig. S7.

The upstream secondary binding site of UAF. (**A**) DNase I footprint visualized by primer extension using the template strand from position −214 of the 35S rRNA promoter. From left to right, Sanger sequencing reactions (T, G, C, A), untreated DNA (−), free DNA treated with 0.36 U and 0.72 U DNase I (DNA), DNA bound to UAF (4, 8 μM), UAF and TBP (4, 8 μM, 1:1 ratio), and TBP (4, 8, 16, 32 μM), treated with 0.72 U DNase I. Sequencing lanes are labelled according to the non-template strand. Bars indicate protection. Asterisks indicate increased sensitivity to DNase. (**B**) Filter binding assays. UAF (0.5 nM – 5 μM) or TBP (0.5 nM – 60 μM) was titrated against radioactive DNA spanning positions −110 to −40 (orange, $DNA^{-110/-40}$) and −180 to −110 (blue, $DNA^{-180/-110}$) twice on separate days. Hill equations with a Hill coefficient of 1 were fitted. Fitted K_d values for UAF are indicated. Shaded regions represent the 95% confidence intervals of the fit, which correspond to K_d values of 16–31 nM and 64–127 nM for $DNA^{-180/-110}$ and $DNA^{-110/-40}$ in repeat 1, respectively; and 13–24 nM and 53–162 nM in repeat 2, respectively. (**C**) Competition filter binding assays. Labelled $DNA^{-180/-110}$ (blue) bound to UAF (100 nM) was challenged with unlabelled $DNA^{-110/-40}$, and labelled $DNA^{-110/-40}$ (orange) bound to UAF (60 nM) was challenged with unlabelled $DNA^{-180/-110}$. A one-site competition model (*60*) was fitted to each series. The experiment was performed twice on separate days.

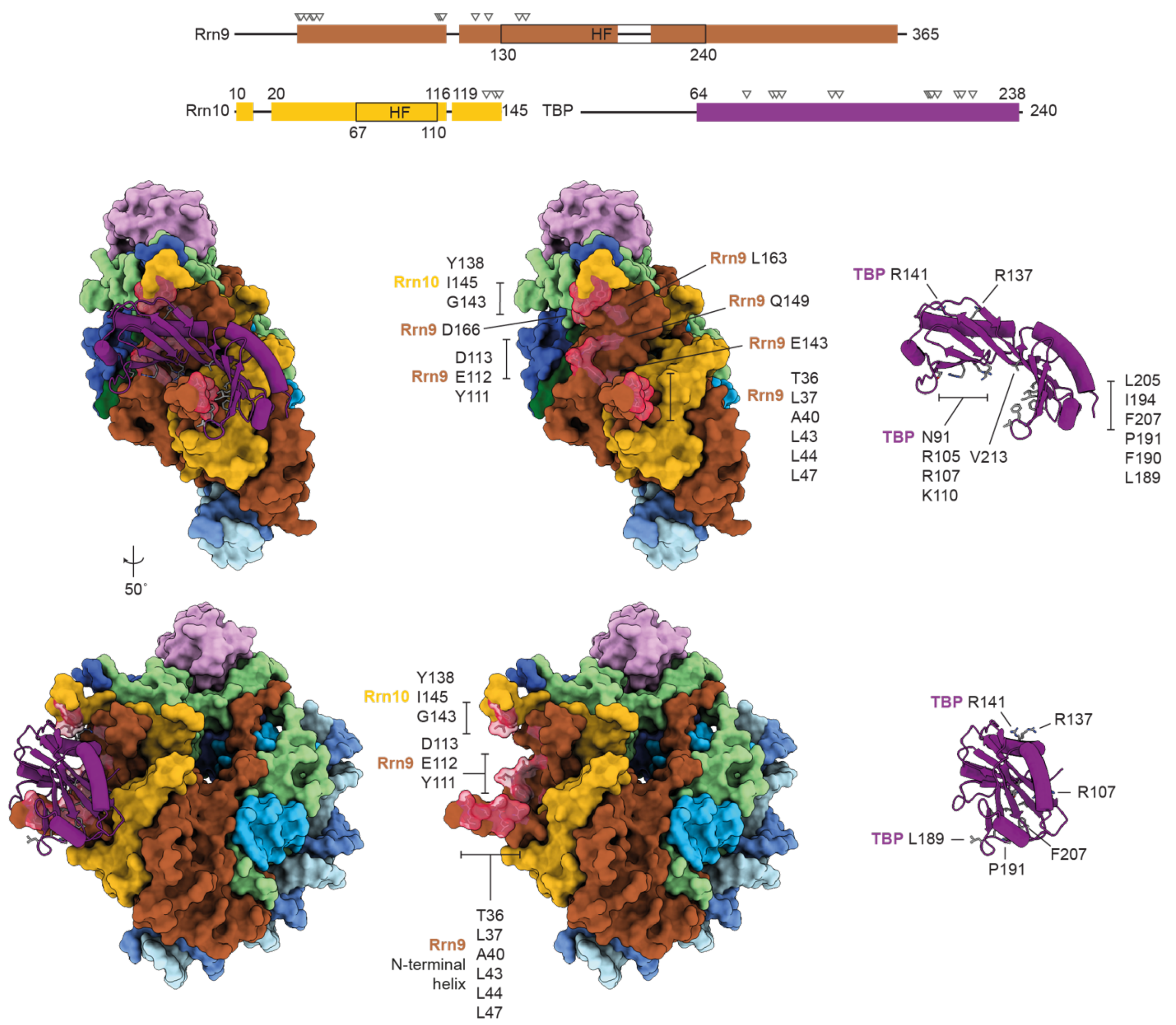

Fig. S8.

Interactions of UAF with TBP. UAF residues interacting with TBP are highlighted in red, and indicated with triangles in the domain diagram. Interacting TBP residues are also indicated.

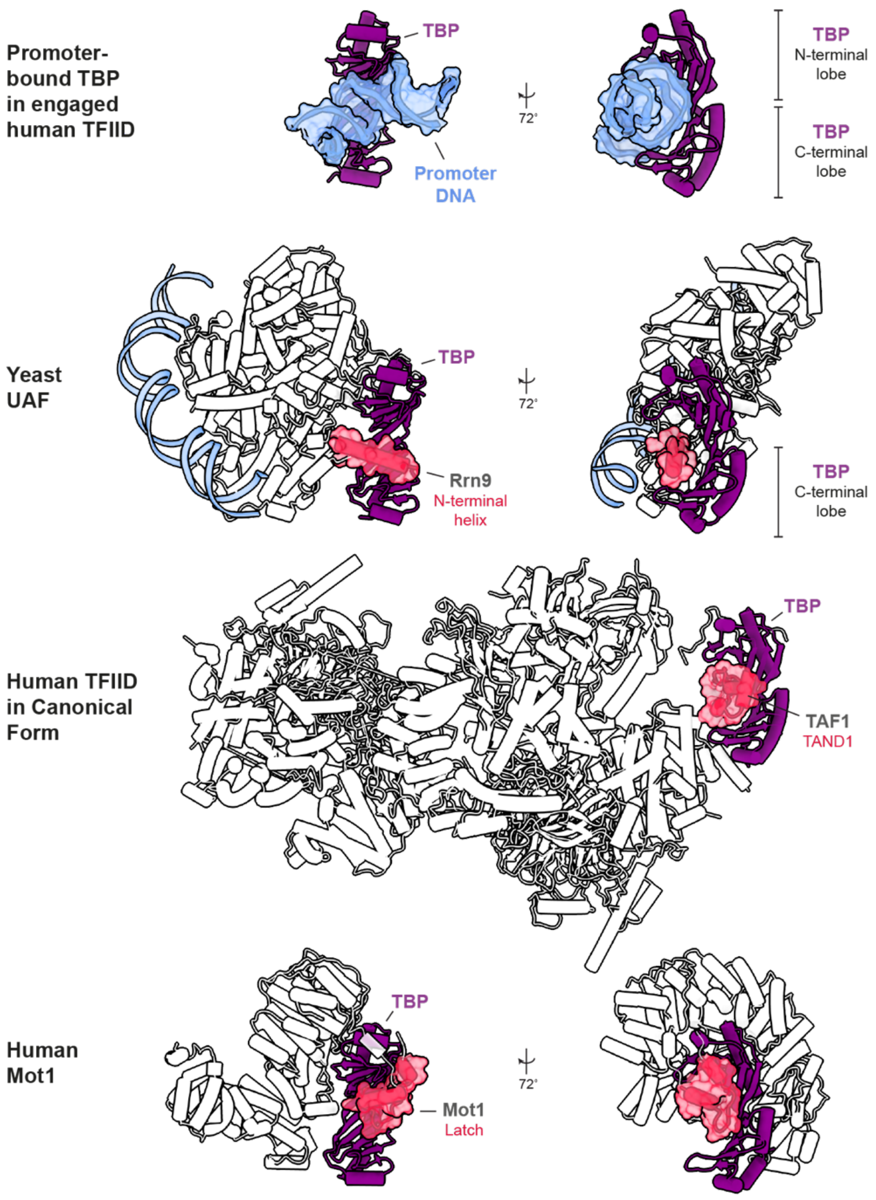

Fig. S9.

Occlusion of the TBP DNA-binding pocket. TBP in engaged human TFIID in initial loading state on DNA (PDB 7EGD) is shown on top. The solvent-excluded surface of the TBP-bound DNA is displayed in blue. Depicted below are the UAF-TBP-DNA complex, human TFIID in canonical form (PDB 6MZL), and Mot1-TBP (PDB 3OC3), aligned according to TBP. Indicated in red are the N-terminal helix of UAF subunit Rrn9, the TAF1 N-terminal domain 1 (TAND1) of TFIID subunit TAF1, and the TBP-occluding latch of Mot1. The solvent-excluded surfaces of these elements are shown. TBP is denoted in purple.

Table S1.
Cryo-EM data collection and model refinement and validation statistics.

	UAF-TBP-DNA (EMDB-14428) (PDB 7Z0O)
**Data collection and processing**	
Magnification	130 000
Voltage (kV)	300
Electron exposure ($e^-/Å^2$)	49.4–49.6
Defocus range (μm)	0.9–1.9
Pixel size (Å)	0.645
Symmetry imposed	C1
Initial particle images (no.)	1 479 233
Final particle images (no.)	193 226
Map resolution (Å)	2.8
FSC threshold	0.143
Map resolution range (Å)	2.5–4.5
**Refinement**	
Initial model used (PDB code)	1id3, 1ngm
Model resolution (Å)	3.1
FSC threshold	0.500
Model composition	
Non-hydrogen atoms	11 866
Protein residues	1 256
Nucleotides	78
B factors ($Å^2$)	
Protein (min/max/mean)	62.9/274.5/99.8
Nucleotide (min/max/mean)	81.9/166.5/109.6
R.m.s. deviations	
Bond lengths (Å)	0.004
Bond angles (°)	0.665
Validation	
MolProbity score	1.45
Clashscore	6.11
Poor rotamers (%)	1.42
Ramachandran plot	
Favored (%)	98.3
Allowed (%)	1.7
Disallowed (%)	0.00

Table S2.
Protein-DNA contacts in the determined structure.

Protein					DNA				Distance (Å)
**Subunit**	**Chain**	**Residue**	**Number**	**Atom**	**Chain**	**Base**	**Position**	**Atom**	
Proximal H3	C	LYS	38	NZ	T	DT	−86	OP2	3.7
Proximal H3	C	HIS	40	N	T	DT	−85	OP1	2.5
Proximal H3	C	TYR	42	N	T	DA	−84	OP1	3.1
Proximal H3	C	LYS	43	NZ	T	DT	−83	OP1	2.6
Proximal H3	C	ARG	41	NH1	N	DA	−80	OP1	3.0
Proximal H3	C	ARG	41	NH2	N	DA	−80	OP1	3.1
Proximal H3	C	ARG	54	NH1	T	DC	−75	OP1	3.2
Proximal H3	C	ARG	54	NH2	T	DC	−75	OP2	3.0
Proximal H3	C	ARG	53	NH1	T	DT	−74	OP2	2.6
Rrn5	D	ARG	189	NH2	N	DA	−81	O3'	2.9
Rrn5	D	ARG	288	NE	N	DT	−70	OP1	3.2
Rrn5	D	ARG	288	NH2	N	DT	−70	OP1	3.3
Rrn5	D	LYS	285	NZ	N	DA	−69	OP1	3.1
Rrn5	D	LYS	290	N	N	DA	−69	OP2	2.9
Rrn5	D	LYS	290	NZ	N	DG	−68	OP2	3.0
Rrn9	E	ARG	295	NH2	T	DA	−65	O4'	2.5
Rrn9	E	THR	294	OG1	N	DA	−61	OP1	2.4
Rrn9	E	ARG	309	NH1	N	DA	−61	OP2	2.6
Rrn9	E	ARG	309	NH2	N	DA	−61	OP2	2.7
Rrn9	E	LYS	306	NZ	N	DG	−59	OP2	3.2
Rrn9	E	LYS	308	NZ	N	DG	−57	O6	3.2
Rrn9	E	SER	312	N	T	DT	−56	OP1	3.1
Rrn9	E	TYR	79	OH	T	DC	−55	OP1	2.3
Rrn9	E	LYS	86	NZ	T	DC	−54	OP2	3.5

REFERENCES AND NOTES

1. B. A. Knutson, S. Hahn, TFIIB-related factors in RNA polymerase I transcription. *Biochim. Biophys. Acta* **1829**, 265–273 (2013).

2. L. Vu, I. Siddiqi, B. S. Lee, C. A. Josaitis, M. Nomura, RNA polymerase switch in transcription of yeast rDNA: Role of transcription factor UAF (upstream activation factor) in silencing rDNA transcription by RNA polymerase II. *Proc. Natl. Acad. Sci. U.S.A.* **96**, 4390–4395 (1999).

3. M. Oakes, I. Siddiqi, L. Vu, J. Aris, M. Nomura, Transcription factor UAF, expansion and contraction of ribosomal DNA (rDNA) repeats, and RNA polymerase switch in transcription of yeast rDNA. *Mol. Cell. Biol.* **19**, 8559–8569 (1999).

4. I. Siddiqi, J. Keener, L. Vu, M. Nomura, Role of TATA binding protein (TBP) in yeast ribosomal DNA transcription by RNA polymerase I: Defects in the dual functions of transcription factor UAF cannot be suppressed by TBP. *Mol. Cell. Biol.* **21**, 2292–2297 (2001).

5. H. Goetze, M. Wittner, S. Hamperl, M. Hondele, K. Merz, U. Stoeckl, J. Griesenbeck, Alternative chromatin structures of the 35S rRNA genes in *Saccharomyces cerevisiae* provide a molecular basis for the selective recruitment of RNA polymerases I and II. *Mol. Cell. Biol.* **30**, 2028–2045 (2010).

6. J. Keener, C. A. Josaitis, J. A. Dodd, M. Nomura, Reconstitution of yeast RNA polymerase I transcription in vitro from purified components: TATA-binding protein is not required for basal transcription. *J. Biol. Chem.* **273**, 33795–33802 (1998).

7. K. Merz, M. Hondele, H. Goetze, K. Gmelch, U. Stoeckl, J. Griesenbeck, Actively transcribed rRNA genes in *S. cerevisiae* are organized in a specialized chromatin associated with the high-mobility group protein Hmo1 and are largely devoid of histone molecules. *Genes Dev.* **22**, 1190–1204 (2008).

8. T. Iida, T. Kobayashi, RNA polymerase I activators count and adjust ribosomal RNA gene copy number. *Mol. Cell* **73**, 645–654.e13 (2019).

9. M. J. Rossi, P. K. Kuntala, W. K. M. Lai, N. Yamada, N. Badjatia, C. Mittal, G. Kuzu, K. Bocklund, N. P. Farrell, T. R. Blanda, J. D. Mairose, A. V. Basting, K. S. Mistretta, D. J. Rocco, E. S. Perkinson, G. D. Kellogg, S. Mahony, B. F. Pugh, A high-resolution protein architecture of the budding yeast genome. *Nature* **592**, 309–314 (2021).

10. J. S. Steffan, D. A. Keys, J. A. Dodd, M. Nomura, The role of TBP in rDNA transcription by RNA polymerase I in *Saccharomyces cerevisiae*: TBP is required for upstream activation factor-dependent recruitment of core factor. *Genes Dev.* **10**, 2551–2563 (1996).

11. X. Chen, Y. Qi, Z. Wu, X. Wang, J. Li, D. Zhao, H. Hou, Y. Li, Z. Yu, W. Liu, M. Wang, Y. Ren, Z. Li, H. Yang, Y. Xu, Structural insights into preinitiation complex assembly on core promoters. *Science* **372**, eaba8490 (2021).

12. D. A. Herbst, M. N. Esbin, R. K. Louder, C. Dugast-Darzacq, G. M. Dailey, Q. Fang, X. Darzacq, R. Tjian, E. Nogales, Structure of the human SAGA coactivator complex. *Nat. Struct. Mol. Biol.* **28**, 989–996 (2021).

13. M. K. Vorländer, H. Khatter, R. Wetzel, W. J. H. Hagen, C. W. Müller, Molecular mechanism of promoter opening by RNA polymerase III. *Nature* **553**, 295–300 (2018).

14. Y. Sadian, F. Baudin, L. Tafur, B. Murciano, R. Wetzel, F. Weis, C. W. Müller, Molecular insight into RNA polymerase I promoter recognition and promoter melting. *Nat. Commun.* **10**, 5543 (2019).

15. M. Pilsl, C. Engel, Structural basis of RNA polymerase I pre-initiation complex formation and promoter melting. *Nat. Commun.* **11**, 1206 (2020).

16. Y. Han, C. Yan, T. H. D. Nguyen, A. J. Jackobel, I. Ivanov, B. A. Knutson, Y. He, Structural mechanism of ATP-independent transcription initiation by RNA polymerase I. *eLife* **6**, e27414 (2017).

17. D. A. Keys, B. S. Lee, J. A. Dodd, T. T. Nguyen, L. Vu, E. Fantino, L. M. Burson, Y. Nogi, M. Nomura, Multiprotein transcription factor UAF interacts with the upstream element of the yeast RNA polymerase I promoter and forms a stable preinitiation complex. *Genes Dev.* **10**, 887–903 (1996).

18. J. Keener, J. A. Dodd, D. Lalo, M. Nomura, Histones H3 and H4 are components of upstream activation factor required for the high-level transcription of yeast rDNA by RNA polymerase I. *Proc. Natl. Acad. Sci. U.S.A.* **94**, 13458–13462 (1997).

19. M. L. Smith, W. Cui, A. J. Jackobel, N. Walker-Kopp, B. A. Knutson, Reconstitution of RNA polymerase I upstream activating factor and the roles of Histones H3 and H4 in complex assembly. *J. Mol. Biol.* **430**, 641–654 (2018).

20. B. A. Knutson, M. L. Smith, A. E. Belkevich, A. M. Fakhouri, Molecular topology of RNA polymerase I upstream activation factor. *Mol. Cell. Biol.* **40**, e00056-20 (2020).

21. A. B. Patel, R. K. Louder, B. J. Greber, S. Grünberg, J. Luo, J. Fang, Y. Liu, J. Ranish, S. Hahn, E. Nogales, Structure of human TFIID and mechanism of TBP loading onto promoter DNA. *Science* **362**, eaau8872 (2018).

22. G. Papai, A. Frechard, O. Kolesnikova, C. Crucifix, P. Schultz, A. Ben-Shem, Structure of SAGA and mechanism of TBP deposition on gene promoters. *Nature* **577**, 711–716 (2020).

23. A. Butryn, J. M. Schuller, G. Stoehr, P. Runge-Wollmann, F. Förster, D. T. Auble, K.-P. Hopfner, Structural basis for recognition and remodeling of the TBP:DNA:NC2 complex by Mot1. *eLife* **4**, e07432 (2015).

24. M. Liu, A. Guo, B. Boukhgalter, K. Van Den Huevel, M. Tripp, L. Pape, Characterization of the fission yeast ribosomal DNA binding factor: Components share homology with upstream activating factor and with SWI/SNF subunits. *Nucleic Acids Res.* **30**, 5347–5359 (2002).

25. J. C. Mars, M. Sabourin-Felix, M. G. Tremblay, T. Moss, A deconvolution protocol for ChIP-seq reveals analogous enhancer structures on the mouse and human ribosomal RNA genes. *Genes Genomes Genet.* **8**, 303–314 (2018).

26. C. L. White, R. K. Suto, K. Luger, Structure of the yeast nucleosome core particle reveals fundamental changes in internucleosome interactions. *EMBO J.* **20**, 5207–5218 (2001).

27. T. J. Richmond, C. A. Davey, The structure of DNA in the nucleosome core. *Nature* **423**, 145–150 (2003).

28. T. Dršata, N. Špačková, P. Jurečka, M. Zgarbová, J. Šponer, F. Lankaš, Mechanical properties of symmetric and asymmetric DNA A-tracts: Implications for looping and nucleosome positioning. *Nucleic Acids Res.* **42**, 7383–7394 (2014).

29. R. K. Louder, Y. He, J. R. López-Blanco, J. Fang, P. Chacón, E. Nogales, Structure of promoter-bound TFIID and model of human pre-initiation complex assembly. *Nature* **531**, 604–609 (2016).

30. K. Kamada, F. Shu, H. Chen, S. Malik, G. Stelzer, R. G. Roeder, M. Meisterernst, S. K. Burley, Crystal structure of negative cofactor 2 recognizing the TBP-DNA transcription complex. *Cell* **106**, 71–81 (2001).

31. R. A. Coleman, B. F. Pugh, Evidence for functional binding and stable sliding of the TATA binding protein on nonspecific DNA. *J. Biol. Chem.* **270**, 13850–13859 (1995).

32. C. N. J. Ravarani, T. Flock, S. Chavali, M. Anandapadamanaban, M. M. Babu, S. Balaji, Molecular determinants underlying functional innovations of TBP and their impact on transcription initiation. *Nat. Commun.* **11**, 2384 (2020).

33. P. Wollmann, S. Cui, R. Viswanathan, O. Berninghausen, M. N. Wells, M. Moldt, G. Witte, A. Butryn, P. Wendler, R. Beckmann, D. T. Auble, K. P. Hopfner, Structure and mechanism of the Swi2/Snf2 remodeller Mot1 in complex with its substrate TBP. *Nature* **475**, 403–407 (2011).

34. B. A. Knutson, J. Luo, J. Ranish, S. Hahn, Architecture of the *Saccharomyces cerevisiae* RNA polymerase I core factor complex. *Nat. Struct. Mol. Biol.* **21**, 810–816 (2014).

35. C. W. Garvie, C. Wolberger, Recognition of specific DNA sequences. *Mol. Cell* **8**, 937–946 (2001).

36. A. Huber, B. Bodenmiller, A. Uotila, M. Stahl, S. Wanka, B. Gerrits, R. Aebersold, R. Loewith, Characterization of the rapamycin-sensitive phosphoproteome reveals that Sch9 is a central coordinator of protein synthesis. *Genes Dev.* **23**, 1929–1943 (2009).

37. A. Soulard, A. Cremonesi, S. Moes, F. Schütz, P. Jenö, M. N. Hall, The rapamycin-sensitive phosphoproteome reveals that TOR controls protein kinase A toward some but not all substrates. *Mol. Biol. Cell* **21**, 3475–3486 (2010).

38. C. P. Albuquerque, M. B. Smolka, S. H. Payne, V. Bafna, J. Eng, H. Zhou, A multidimensional chromatography technology for in-depth phosphoproteome analysis. *Mol. Cell. Proteomics* **7**, 1389–1396 (2008).

39. H. S. Rhee, B. F. Pugh, Genome-wide structure and organization of eukaryotic pre-initiation complexes. *Nature* **483**, 295–301 (2012).

40. G. E. Ghanim, A. J. Fountain, A. M. M. van Roon, R. Rangan, R. Das, K. Collins, T. H. D. Nguyen, Structure of human telomerase holoenzyme with bound telomeric DNA. *Nature* **593**, 449–453 (2021).

41. D. N. Mastronarde, Automated electron microscope tomography using robust prediction of specimen movements. *J. Struct. Biol.* **152**, 36–51 (2005).

42. A. Punjani, J. L. Rubinstein, D. J. Fleet, M. A. Brubaker, CryoSPARC: Algorithms for rapid unsupervised cryo-EM structure determination. *Nat. Methods* **14**, 290–296 (2017).

43. S. H. W. Scheres, A bayesian view on cryo-EM structure determination. *J. Mol. Biol.* **415**, 406–418 (2012).

44. A. Casañal, B. Lohkamp, P. Emsley, Current developments in Coot for macromolecular model building of electron cryo-microscopy and crystallographic data. *Protein Sci.* **29**, 1069–1078 (2020).

45. R. Sanchez-Garcia, J. Gomez-Blanco, A. Cuervo, J. M. Carazo, C. O. S. Sorzano, J. Vargas, DeepEMhancer: A deep learning solution for cryo-EM volume post-processing. *Commun. Biol.* **4**, 874 (2021).

46. Z. S. Juo, G. A. Kassavetis, J. Wang, E. P. Geiduschek, P. B. Sigler, Crystal structure of a transcription factor IIIB core interface ternary complex. *Nature* **422**, 534–539 (2003).

47. M. Kowiel, D. Brzezinski, M. Gilski, M. Jaskolski, Conformation-dependent restraints for polynucleotides: The sugar moiety. *Nucleic Acids Res.* **48**, 962–973 (2020).

48. M. Gilski, J. Zhao, M. Kowiel, D. Brzezinski, D. H. Turner, M. Jaskolski, Accurate geometrical restraints for Watson–Crick base pairs. *Acta Crystallogr. Sect. B* **75**, 235–245 (2019).

49. P. V. Afonine, B. K. Poon, R. J. Read, O. V. Sobolev, T. C. Terwilliger, A. Urzhumtsev, P. D. Adams, Real-space refinement in PHENIX for cryo-EM and crystallography. *Acta Crystallogr. Sect. D* **74**, 531–544 (2018).

50. C. J. Williams, J. J. Headd, N. W. Moriarty, M. G. Prisant, L. L. Videau, L. N. Deis, V. Verma, D. A. Keedy, B. J. Hintze, V. B. Chen, S. Jain, S. M. Lewis, W. B. Arendall, J. Snoeyink, P. D. Adams, S. C. Lovell, J. S. Richardson, D. C. Richardson, MolProbity: More and better reference data for improved all-atom structure validation. *Protein Sci.* **27**, 293–315 (2018).

51. C. Blanchet, M. Pasi, K. Zakrzewska, R. Lavery, CURVES+ web server for analyzing and visualizing the helical, backbone and groove parameters of nucleic acid structures. *Nucleic Acids Res.* **39**, W68–W73 (2011).

52. E. F. Pettersen, T. D. Goddard, C. C. Huang, E. C. Meng, G. S. Couch, T. I. Croll, J. H. Morris, T. E. Ferrin, UCSF ChimeraX: Structure visualization for researchers, educators, and developers. *Protein Sci.* **30**, 70–82 (2021).

53. E. Jurrus, D. Engel, K. Star, K. Monson, J. Brandi, L. E. Felberg, D. H. Brookes, L. Wilson, J. Chen, K. Liles, M. Chun, P. Li, D. W. Gohara, T. Dolinsky, R. Konecny, D. R. Koes, J. E. Nielsen, T. Head-Gordon, W. Geng, R. Krasny, G. W. Wei, M. J. Holst, J. A. McCammon, N. A. Baker, Improvements to the APBS biomolecular solvation software suite. *Protein Sci.* **27**, 112–128 (2018).

54. E. Krissinel, K. Henrick, Inference of macromolecular assemblies from crystalline state. *J. Mol. Biol.* **372**, 774–797 (2007).

55. E. Lesage, J. Perez-Fernandez, S. Queille, C. Dez, O. Gadal, M. Kwapisz, Non-coding, rnapii-dependent transcription at the promoters of rrna genes regulates their chromatin state in *S. cerevisiae*. *Non Coding RNA* **7**, 41 (2021).

56. S. Schilbach, S. Aibara, C. Dienemann, F. Grabbe, P. Cramer, Structure of RNA polymerase II pre-initiation complex at 2.9 Å defines initial DNA opening. *Cell* **184**, 4064–4072.e28 (2021).

57. P. J. Willy, R. Kobayashi, J. T. Kadonaga, A basal transcription factor that activates or represses transcription. *Science* **290**, 982–984 (2000).

58. Y. Cang, G. Prelich, Direct stimulation of transcription by negative cofactor 2 (NC2) through TATA-binding protein (TBP). *Proc. Natl. Acad. Sci. U.S.A.* **99**, 12727–12732 (2002).

59. E. Y. D. Chua, D. Vasudevan, G. E. Davey, B. Wu, C. A. Davey, The mechanics behind DNA sequence-dependent properties of the nucleosome. *Nucleic Acids Res.* **40**, 6338–6352 (2012).

60. C. Yung-Chi, W. H. Prusoff, Relationship between the inhibition constant (KI) and the concentration of inhibitor which causes 50 per cent inhibition (I50) of an enzymatic reaction. *Biochem. Pharmacol.* **22**, 3099–3108 (1973).
